# Tuning the Brain: How Acoustic Energies Modulate Neural and Glial Phenotypes

**DOI:** 10.3390/ijms27219481

**Published:** 2026-10-23

**Authors:** Giovanni Luca Cipriano, Emilio Agostinelli, Alessia Floramo, Veronica Argento, Sara Spinelli, Marco Calabrò, Ivan Anchesi

**Affiliations:** 1 IRCCS Centro Neurolesi “Bonino-Pulejo”, Via Provinciale Palermo, Contrada Casazza, 98124 Messina, Italy; giovanniluca.cipriano@irccsme.it (G.L.C.); emilio.agostinelli@irccsme.it (E.A.); alessia.floramo@irccsme.it (A.F.); veronica.argento@irccsme.it (V.A.); ivan.anchesi@irccsme.it (I.A.); 2 Department of Biomedical and Dental Sciences and Morpho-Functional Imaging—BIOMORF, University of Messina, 98125 Messina, Italy; mcalabro@unime.it

**Keywords:** mechanotransduction, sonobiology, low-intensity pulsed ultrasound (LIPUS), neuroregeneration, glial plasticity, Piezo1 channel, acoustic neuromodulation, sonogenetics, microglial polarization, extracorporeal shockwave therapy (ESWT), acoustic dosimetry, evidence appraisal

## Abstract

The traditional view of neuronal communication is shifting toward a mechanobiological paradigm. This review examines how mechanical energy from diverse acoustic stimuli—audible sound and whole-body or vibrotactile vibration, low-intensity pulsed ultrasound (LIPUS), low-intensity focused ultrasound (LIFU), high-intensity focused ultrasound (HIFU), and extracorporeal shockwave therapy (ESWT)—translates into biochemical signals. Evidence is stratified throughout by model system (in vitro, animal in vivo, human) and by reported exposure dose, and sensory-pathway or network-level effects of audible sound are kept distinct from direct cellular mechanotransduction. Acoustic forces are directly detected by primary mechanosensitive ion channels, such as Piezo1 and TRPV4, and experimental evidence links this mechanotransduction to neurotrophic signaling (mechanistic target of rapamycin (mTOR); p38 mitogen-activated protein kinase (MAPK); brain-derived neurotrophic factor (BDNF)) that supports neurite outgrowth and structural repair, whereas a continuous signaling axis running from the cytoskeleton through the linker of nucleoskeleton and cytoskeleton (LINC) complex to epigenetic regulation remains a mechanistic hypothesis that has not been demonstrated in any acoustically stimulated neural cell. Furthermore, glial cells act as active mechanosensors, and emerging evidence suggests that acoustic stimulation can promote microglial polarization toward neuroprotective M2 phenotypes and may support Schwann cell-mediated peripheral nerve regeneration. We discuss the emerging concept of “acoustic pharmacology,” in which specific waveforms and frequencies dictate distinct therapeutic outcomes. From targeted amyloid clearance in Alzheimer’s disease to the non-invasive neural circuit control offered by sonogenetics, acoustic stimulation is a tunable but still predominantly preclinical frontier in regenerative medicine and neuromodulation: of the 144 sources cited here, seven report human studies, and the only randomized, sham-controlled evidence with a clinical endpoint concerns ablative high-intensity focused ultrasound; for the non-ablative neuromodulatory, neuroprotective, and regenerative regimes that are the subject of this review, no such trial is present in this evidence base.

## 1. Introduction

### 1.1. The Mechanobiological Shift

The traditional view of neuronal communication relies on the Hodgkin and Huxley (HH) model, which treats the action potential as a purely electrical phenomenon driven by voltage- and time-dependent opening and closure of ion channels, allowing passive flow of Na^+^ and K^+^ ions across the axonal membrane [1]. In this framework, the axonal membrane is conceptualized as an electronic circuit—a capacitor with ion channels as resistors—and the action potential propagates via local current flow and diffusion of cations in the neuronal cytoplasm [1,2]. However, an emerging alternative describes the action potential as a non-linear acoustic pulse propagating within a biological interface near a phase transition, a model introduced by Heimburg and Jackson and built on thermodynamic principles [1,2]. Biophysical models have proposed that electrical activity may be accompanied by coupled mechanical and thermodynamic changes in neuronal membranes and lipid interfaces [3,4,5]. These approaches provide complementary perspectives on neuronal signaling rather than an established replacement for its electrophysiological description. Experiments on protein-free lipid bilayers have demonstrated ultrasound-induced membrane displacement and changes in capacitance [6].

### 1.2. Physical Principles

The biophysical coupling of acoustic energy to biological tissues is governed by distinct mechanical phenomena [7]. Acoustic streaming can enhance fluid mixing and convective transport [8]. Acoustic radiation force (ARF), the time-averaged force exerted on an absorbing or reflecting medium by momentum transfer from a propagating wave, transfers momentum from the propagating ultrasound wave directly to the tissue, causing localized mechanical displacement [8]. Additionally, when continuous intensity gradients exist in a fluid medium, the fluid within the ultrasound beam acquires momentum in the direction of wave propagation, creating a compensating circulatory flow known as acoustic streaming [9]. This acoustic streaming imparts shear stress along cellular boundaries and promotes the enhanced diffusion of molecules across the microenvironment [10]. Concurrently, cavitation describes the formation of vapor cavities when a soft biological system is subjected to a transient pressure drop below its saturated vapor pressure [11]. Inertial cavitation involves unstable bubble expansion followed by violent collapse, producing a highly localized energy release—often manifesting as microjetting—that can mechanically ablate nearby cellular structures [12]. In contrast, stable cavitation is characterized by lower-amplitude, sustained microbubble oscillations around an equilibrium radius. These oscillations generate localized microstreaming and increased shear stress at the cell membrane, thereby promoting reversible sonoporation without causing catastrophic structural damage. Sonoporation—the transient permeabilization of the plasma membrane by oscillating or collapsing microbubbles—is mechanistically distinct from gating of endogenous mechanosensitive channels: it admits solutes and ions non-selectively and is accompanied by dye influx and membrane-repair signatures, so ultrasound-evoked Ca^2+^ signals obtained in the presence of microbubbles cannot be attributed to channel gating without additional controls [13]. Absorption-driven heating constitutes a third, separable category of bioeffect and underlies both high-intensity ablation and engineered thermosensitive actuators such as TRPV1 in sonothermogenetics [14,15]. Throughout this review, the mechanical, cavitational, and thermal routes are kept distinct because they differ in dose dependence, in reversibility, and in safety profile [16,17].

## 2. The Molecular Code of Mechanotransduction

The translation of acoustic and mechanical stimuli into biochemical signals relies fundamentally on specialized transmembrane ion channels. The Piezo family, particularly Piezo1 and Piezo2, operates as a primary mechanotransducer. Structurally, the Piezo1 channel homotrimerizes into a 0.9 MDa propeller-like architecture featuring nonplanar transmembrane blades curved into a “nano-bowl” shape [18]. Upon activation by low-intensity, low-frequency ultrasound, Piezo1 functions as a non-selective cation channel that mediates Ca^2+^ influx [19]. In neurons and in heterologous systems, this attribution rests on Piezo1 knockdown and on GsMTx-4 pharmacology, which attenuate but do not abolish the ultrasound-evoked Ca^2+^ response; Piezo1 is therefore contributory rather than solely responsible, and a residual Piezo1-independent component is consistently observed [20]. This intracellular calcium accumulation initiates downstream signaling cascades, including the activation of the calmodulin kinase (CaMKII) pathway [21]. Piezo2 shares similar mechanosensitive properties but serves a distinctly different acoustic physiological function, acting critically within cochlear outer hair cells to coordinate hair-bundle mechanotransduction for ultrasonic hearing in murine models [18]. Alongside Piezo channels, members of the transient receptor potential (TRP) family serve as multimodal biosensors. TRPV4 participates in mechanically evoked Ca^2+^ signaling, including responses to fluid shear stress [22]. The clearest genetic evidence that an identified channel mediates an acoustic effect in neural tissue comes from astrocytes: astrocyte-specific deletion of Trpa1 abolishes the Ca^2+^ response to low-intensity, low-frequency ultrasound and the consequent neuronal activation, whereas pharmacological blockade alone would not have distinguished this from off-target effects [23]. In murine kidney and skeletal muscle models—that is, outside the nervous system—pulsed focused ultrasound (pFUS) has been shown to engage TRPC1-dependent Na^+^ influx upstream of voltage-gated Ca^2+^ channels [24,25]. In contrast to the depolarizing calcium channels, two-pore-domain potassium (K2P) channels are recruited to modulate and balance neuronal excitability. The TREK subgroup, encompassing TREK-1, TREK-2, and TRAAK, is highly sensitive to mechanical force at the cellular membrane [26,27]. Activation of the TRAAK channel by membrane tension generates a hyperpolarizing current [28]. Activation of mechanosensitive K2P channels generates outward K+ currents that can reduce neuronal excitability [28,29].

### Sources and Interpretation of Acoustically Evoked Ca^2+^ Signals

Because almost every downstream effect proposed in this field is described as Ca^2+^-dependent, it is necessary to state explicitly which Ca^2+^ source has actually been identified in each model, and by which method. Three routes must be separated: (i) direct entry through primary mechanosensitive channels (Piezo1, TRPV4, TRPA1, and MscL in engineered systems); (ii) secondary recruitment of voltage-gated Ca^2+^ channels following a depolarizing cation or Na^+^ current; and (iii) release from intracellular stores. The strongest evidence in neural cells is for routes (i) and (ii) operating in series. In cultured cortical neurons, 300 kHz focused ultrasound (ISPPA up to 15 W/cm^2^, measured in free water) evokes a Ca^2+^ rise whose initiation requires mechanosensitive entry and whose magnitude is then amplified by voltage-gated channels, establishing an explicit upstream–downstream order [30]. In kidney and skeletal muscle, pFUS acts through TRPC1-dependent Na^+^ influx upstream of voltage-gated Ca^2+^ channels [24,25]. In astrocytes, cell-type-specific Trpa1 deletion abolishes the response, which is a genetic rather than a pharmacological attribution [23]. In cultured neurons, Piezo1 knockdown and GsMTx-4 only partially reduce the response, so the channel is contributory but not exclusive [20]. By contrast, several widely cited reports in microglia, Schwann cells, and stromal cells rest on Ca^2+^ imaging alone, without genetic deletion, selective pharmacology, or electrophysiological recording; in those models, the responsible conductance should be regarded as unidentified, and we label it as such in Table 1.

Three further distinctions are required before a Ca^2+^ signal can be read as a beneficial signaling event. First, the biological meaning of a Ca^2+^ transient is encoded in its amplitude, duration, frequency, and subcellular localization rather than in its occurrence: brief, spatially restricted elevations drive CaMKII- and CREB-dependent plasticity programs, whereas sustained, global elevation engages calpain activation, mitochondrial Ca^2+^ overload, and stress or death pathways [57]. An increase in intracellular Ca^2+^ is therefore not by itself evidence of action-potential generation, of enhanced transmitter release, or of regeneration, and none of the studies reviewed here that report Ca^2+^ imaging alone can support those inferences. Second, mechanically mediated responses must be separated from sonoporation, in which microbubble-driven permeabilization admits Ca^2+^ non-selectively [13]. Third, they must be separated from inherently thermal mechanisms: sonothermogenetics activates TRPV1 through a focal temperature rise measured by magnetic resonance thermometry rather than through membrane strain and is therefore a thermal paradigm that happens to use ultrasound as the heat source [15].

A complementary, non-acoustic line of evidence supports the underlying premise that targeted mechanical force at the membrane is sufficient to gate neuronal Ca^2+^ entry, and it is instructive precisely because it dissociates force from the acoustic field. Magnetomechanical actuation of cortical neural networks with functionalized magnetic nanoparticles elicits Ca^2+^ influx in which N-type voltage-gated Ca^2+^ channels are implicated [58], and repeated nanomagnetic stimulation restores mechanosensitive channel equilibrium in neural networks [59]; fluorescent magnetic nanoparticles similarly modulate intracellular Ca^2+^ levels in motoneurons [60]. Because these systems deliver force without acoustic pressure fields, they isolate the mechanical component from acoustic streaming, cavitation, and absorption-driven heating, and they indicate that a mechanically gated Ca^2+^ response can recruit voltage-gated channels as a secondary amplifier—the same two-step architecture inferred for ultrasound [30]. They do not, however, establish that acoustic exposure engages the same channels at the pressures and frequencies used therapeutically.

Once mechanical stimuli traverse the plasma membrane, the resulting forces are transmitted through the cytoskeleton, which behaves as a prestressed tensegrity network composed of discrete tensile and compressive elements [61]. In this framework, the cell maintains structural integrity by dynamically balancing internal forces: tensile prestress is generated actively by actomyosin contractility and passively by cytoplasmic swelling and matrix adhesion and is primarily carried by actin microfilaments and intermediate filaments [62]. This continuous tension system is counterbalanced by an interconnected set of compression-bearing microtubules, which act as buckling struts that support part of the contractile load and contribute to cell shape stability and stiffness in accordance with tensegrity model predictions [62].

Cytoskeletal networks transmit mechanical forces to the nucleus, providing a plausible physical route through which acoustically induced cellular deformation could influence nuclear mechanics; however, whether this prestressed architecture actually functions as an efficient, low-loss “intracellular acoustic waveguide” remains to be experimentally established, rather than assumed.

To relay this balanced mechanical energy from the cytoplasm directly to the genome, the cell utilizes the linker of nucleoskeleton and cytoskeleton (LINC) complex [63]. This specialized architecture forms a physical bridge across the nuclear envelope, integrating the external cytoskeletal lattice with the internal nuclear environment [63]. The LINC complex is principally formed by SUN-domain proteins (notably SUN1 and SUN2) embedded in the inner nuclear membrane and KASH-domain proteins (nesprins) embedded in the outer nuclear membrane [64,65]. The C-terminal SUN domains project into the perinuclear space, where trimeric SUN assemblies directly bind the short luminal KASH domains of nesprins, forming the core SUN–KASH bridge across the nuclear envelope [66]. On the cytoplasmic side, nesprins extend into the cytoplasm and couple the nucleus to distinct cytoskeletal systems: nesprin-1 and nesprin-2 bind actin filaments, nesprin-3 binds plectin to link to intermediate filaments, and nesprin-4 and KASH5 recruit microtubule-based motors (kinesin, dynein), thereby connecting the nuclear envelope to actin microfilaments, intermediate filaments, and microtubules [66,67,68]. Mechanotransduction ultimately converges on the nucleus, where forces dynamically remodel chromatin architecture and thereby shape epigenetic regulation [21,69,70].

In the few systems in which it has been tested directly, the transcriptional response to sound is reproducible within a given cell line and depends on the architecture of the acoustic waveform as well as on the mechanical state of the cell; this has been shown in non-neural mammalian lines and has not been replicated in neurons or glia. In terms of impulse specificity, coherent sinusoidal waves (e.g., 440 Hz) act as stable mechanical signals that reproducibly suppress a defined set of mechanosensitive genes in cultured non-neural mammalian cells [31,32], whereas non-sinusoidal waves (square or triangle) at the same frequency often fail to produce identical effects due to their high-frequency harmonic content [31,32]. This mechanical information is channeled toward the genome via the cytoskeletal tensegrity network and the LINC complex [63,68], which have been proposed—but not demonstrated—to act as an intracellular acoustic waveguide that limits energy dissipation; no study has measured acoustic energy transfer along this route in any cell, and the proposal is retained here only as a working hypothesis. Furthermore, mechanical strain physically flattens the nucleus and dilates nuclear pore complexes [71,72], facilitating the selective nuclear import of transcriptional regulators like YAP [71,72], with effects on gene accessibility currently supported for transcriptional modulation over hours, whereas durable long-term mechanical memory remains to be established [31,32].

When extracellular tension is transmitted through the cytoskeleton and LINC complex to the nuclear lamina, it redistributes nuclear envelope tension and reorganizes chromatin anchoring sites at the periphery [73]. In this way, nuclear lamins and lamina-associated domains act as mechanosensitive hubs that couple envelope deformation to large-scale changes in chromatin organization and accessibility [74].

**Figure 1:**
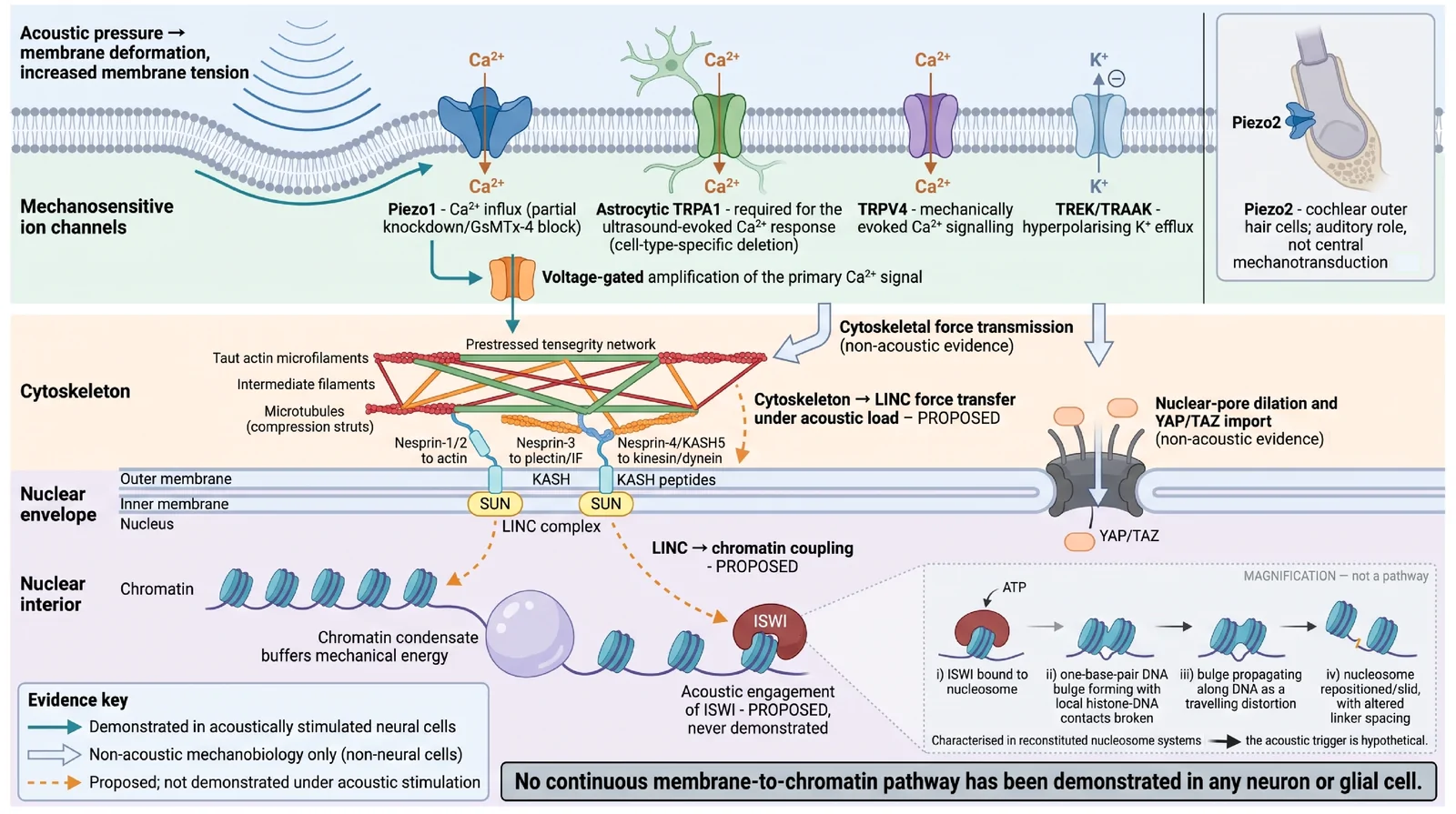
The molecular infrastructure of acoustic mechanotransduction: what is demonstrated and what is proposed. (Main panel) Acoustic pressure deforms the plasma membrane and raises membrane tension. Primary mechanosensors gate in response: the homotrimeric Piezo1 channel mediates Ca^2+^ influx in neurons, although knockdown and GsMTx-4 only partially reduce the response [19,20]; astrocytic TRPA1 is required for the Ca^2+^ response to low-intensity, low-frequency ultrasound and for the downstream neuronal activation [23]; TRPV4 contributes to mechanically evoked Ca^2+^ signaling [22]; and mechanosensitive two-pore-domain K^+^ channels of the TREK/TRAAK family generate opposing hyperpolarizing K^+^ currents [27,29]. Ca^2+^ entering through these primary channels is subsequently amplified by voltage-gated Ca^2+^ channels rather than acting alone [30]. Piezo2, whose characterized acoustic role is in cochlear outer hair cells rather than in central mechanotransduction, is shown separately for contrast [18]. Mechanical tension is then proposed to be transmitted through the cytoskeletal tensegrity network (actin microfilaments, intermediate filaments, and microtubules) to the LINC complex (SUN–KASH bridge) spanning the nuclear envelope [63,66,67,68], and nuclear deformation is proposed to dilate nuclear pores and favor nuclear import of YAP/TAZ [71,72,80]. (Inset) ISWI mechanochemistry as characterized in reconstituted nucleosome systems: the ISWI motor hydrolyzes ATP to generate a one-base-pair DNA bulge that disrupts local histone–DNA contacts; the distortion propagates along nucleosomal DNA and repositions the nucleosome, altering higher-order chromatin topology and gene accessibility [75,76,77,78]. Chromatin can additionally behave as a condensate that buffers mechanical energy [79]. Evidence key. Solid arrows denote steps with direct experimental evidence in acoustically stimulated neural cells: membrane deformation; gating of Piezo1, TRPA1, and TREK/TRAAK channels; Ca^2+^ influx; and voltage-gated amplification [19,20,22,23,27,29,30]. Open arrowheads denote steps supported only by non-acoustic mechanobiology in non-neural cells: cytoskeletal force transmission, LINC-mediated nuclear force transfer, nuclear-pore dilation, and YAP/TAZ import [63,66,67,68,71,72]. Dashed arrows denote proposed connections that have not been demonstrated in any acoustically stimulated neural cell: cytoskeleton→LINC force transfer under acoustic load, LINC→chromatin coupling, and acoustic engagement of ISWI. No element of this figure should be read as a continuous, demonstrated pathway from membrane to chromatin; no such pathway has been shown in a neuron or a glial cell. Dashed rectangular boundaries delimit the enlarged inset and indicate magnification only; they are drawn in a different color and line weight from the pathway arrows, and no leader line in this figure denotes a hypothetical relationship. Figure concepts were derived from [18,19,20,22,23,27,28,30,63,66,71,72,75,76,77,78,80]. Created with BioRender.com. Created in Biorender. Giovanni Luca Cipriano. (2026) https://BioRender.com/jk5wjt9.

At the molecular level, mechanical forces drive three-dimensional chromatin topological remodeling by engaging the imitation switch (ISWI) complex, a specialized motor that converts the chemical energy of ATP hydrolysis into quantized mechanical work. Recent structural evidence reveals that the ISWI motor undergoes precise conformational transitions across its ATPase cycle, which directly distort the DNA strands to generate a full one-base-pair bulge and a localized loss of histone–DNA contacts at the motor-binding site [75]. This process is characterized by sophisticated coordination between nucleosomal sites: DNA is first translocated toward the exit side in discrete 1 bp steps, creating a mechanical strain that, once accumulated, triggers multi-bp compound steps on the entry side to resolve the tension and effectively slide the nucleosome [76,77]. Beyond simple positioning, active ATP hydrolysis is required to fuel the diffusional mobility of ISWI within condensed chromatin environments, preventing the complex from forming static cross-links that would otherwise stiffen the chromatin fiber and blunt the mechanotransductive response [78]. Through this wave-like propagation of DNA distortions and regulated spacing, ISWI reconfigures higher-order chromatin topology, thereby precisely tuning gene accessibility; whether this remodeling occurs in direct response to the physical signature of an acoustic stimulus remains entirely unestablished. This point requires emphasis because the ISWI literature summarized above derives from reconstituted nucleosome biochemistry, single-molecule assays, and cryo-electron microscopy, not from acoustically stimulated cells. The demonstration that ISWI converts ATP hydrolysis into mechanical work on DNA shows that a chromatin remodeler is a molecular motor; it does not show that externally applied acoustic force is transmitted to that motor, nor that it alters its activity. To the best of our knowledge, no study has reported ISWI recruitment, ISWI activity, or nucleosome repositioning following acoustic stimulation of a neuron or glial cell, and the connection drawn in Figure 1 is annotated accordingly.

Under high mechanical or ionic stress, chromatin can undergo liquid–liquid phase separation, behaving locally as a fluid-like condensate that helps dissipate mechanical energy and reduce direct force transmission to DNA, thereby limiting torsional stress and breakage [28]. Condensates formed by chromatin- and IDP-rich phases can mechanically exclude surrounding chromatin, pull genomic loci together, and act as mechano-active filters that reorganize genome topology [79]. Audible and low-intensity acoustic waves act as mechanical stimuli: in several non-neural mammalian cell lines (ST2 bone-marrow stromal cells, C2C12 myoblasts, and NIH3T3 fibroblasts), specific sound patterns rapidly and persistently suppress subsets of mechanosensitive genes via transcriptional control and RNA degradation in a cell-type- and waveform-dependent manner [31]. These responses share pathways with classical mechanotransduction, including focal adhesion and cytoskeleton-dependent signaling, and are thought to act on the open/closed status of chromatin and mechanical memory programs [28,31].

In states of high intracellular tension, compressive forces induce nuclear deformation, which asymmetrically enlarges the diameter of the nuclear pore complexes [28]. This reduction in mechanical restriction facilitates the translocation of YAP into the nucleus. Focused low-intensity pulsed ultrasound (FLIPUS) provides highly specific regulation of this pathway by reducing the phosphorylation of YAP at serine 127 [80]. Because phosphorylation at this site normally sequesters YAP in the cytosol, FLIPUS-induced dephosphorylation permits active YAP to accumulate within the nucleus. Once inside the nucleus, YAP binds to TEAD transcription factors, a response that, in that study, followed focused low-intensity pulsed ultrasound exposure of murine mesenchymal precursors and was accompanied by transcription of genes regulating actin nucleation, cytokinesis, and cell-cycle progression, and hence by proliferation; more broadly, YAP/TEAD signaling regulates proliferative and regenerative gene programs across cell types [80,81]. FLIPUS also illustrates a nomenclature point developed in Section 3.1: focusing and intensity are independent parameters, so a focused field can be delivered at LIPUS intensities and the labels “LIPUS” and “focused ultrasound” denote overlapping rather than mutually exclusive classes.

Taken together, these observations outline a candidate relay—from primary plasma membrane sensors, through the cytoskeletal tensegrity network, to the nucleus—that could map extracellular acoustic cues onto epigenetic and transcriptional outputs (Figure 1). The limits of this construction should be stated explicitly because they differ from step to step. The membrane-sensor step is supported by direct experiments in acoustically stimulated neural cells, including genetic deletion in astrocytes [23] and partial pharmacological and knockdown evidence in neurons [20,30]. The cytoskeleton-to-LINC and LINC-to-chromatin steps are supported by mechanobiology that uses substrate strain, micropipette aspiration, or magnetic tweezers in non-neural cells [63,66,71,72], not acoustic exposure. The chromatin-remodeling step is supported by in vitro biochemistry on reconstituted nucleosomes [75,76,77,78]. No single study has demonstrated the full axis in an acoustically stimulated neuron or glial cell. The axis is therefore presented here as a hypothesis that organizes an otherwise disconnected literature, and not as an established pathway; wherever it is invoked in later sections, the level of evidence available for each link is restated.

## 3. Acoustic Energies and Therapeutic Regimes

Acoustic neuromodulation strategies can be stratified by their distinct energy profiles and resultant tissue interactions. Distinct acoustic energies behave, in this respect, like distinct pharmacological agents: the biological effect depends on the exposure parameters rather than on the use of sound as such, and the same transducer can produce opposite outcomes at different intensities, duty cycles, or pulse-repetition frequencies [7,17,82]. Acoustic interventions can therefore be framed heuristically as an “acoustic pharmacology”, in which waveform, carrier frequency, pulse-repetition frequency, duty cycle, intensity, and focusing jointly determine the mechanism engaged and the plausible therapeutic niche [7,83,84]. This framing is used here as an organizing metaphor and not as a claim of pharmacological equivalence: unlike a drug, an acoustic field has no molecular target of its own and acts only through the mechanical, cavitational, and thermal consequences of energy deposition in tissue, so its specificity is spatial and biophysical rather than molecular [17]. The exposure parameters, model systems, and outcomes of the representative studies discussed below are tabulated in Table 1.

### 3.1. Dosimetry, Exposure Metrics, and Nomenclature

Comparison across studies is possible only if exposure is reported in commensurable units, and much of the apparent inconsistency in this field is dosimetric rather than biological. Ultrasound intensity may be quoted as spatial-peak pulse-average (ISPPA), spatial-peak temporal-average (ISPTA), spatial-average temporal-average (ISATA), or as peak negative pressure, and these quantities are not interconvertible without the duty cycle and the beam profile. The difference is large: in a transcranial protocol using a 2 MHz carrier pulsed at a 40 Hz pulse-repetition frequency with 400 µs pulses, an ISPPA of 190 W/cm^2^ corresponds to an ISPTA of only 3.0 W/cm^2^ [43]. Values are also commonly reported as measured in free water with a calibrated hydrophone, whereas the quantity relevant to biology is the in situ value after attenuation, scattering, and aberration by intervening tissue; for transcranial exposure, the skull attenuates and defocuses the beam in a frequency-dependent and donor-specific manner, so free-water and in situ figures can differ substantially [85]. Throughout this review, we report the metric exactly as given by the original study and annotate it as free-water or in situ only where the source states which was measured; where a source does not specify a parameter, the corresponding entry in Table 1 is marked “NR” (not reported) and has not been imputed. Parameters for every study in Table 1 were re-extracted from the full text and, where available, the supplementary material of the primary source rather than from the abstract. We make no claim that incomplete dosimetric reporting is characteristic of this literature, and the density of “NR” entries in Table 1 should not be read as such a finding: several of the most frequently cited studies here report their exposures in considerable detail, in some cases across several distinct configurations within a single paper, or as a full factorial exploration of frequency, intensity, duty cycle, and exposure time [23,39,48,49,50]. The obstacle to synthesis is not that exposures go unreported but that they are reported in metrics that cannot be converted into one another without the duty cycle and the beam profile, that the free-water or in situ basis of a stated value is often left implicit, and that a single paper may use different exposures for its in vitro, electrophysiological, and behavioral experiments without the difference being evident outside the Methods section. For shockwaves, the conventional dose descriptors are energy flux density (EFD, mJ/mm^2^) and the number of impulses per session, together with the session interval and whether the device is focused or radial. For audible sound and vibration, the relevant descriptors are sound pressure level referenced to 20 µPa (or the pressure in Pa) and, for vibration, the displacement amplitude or acceleration together with the drive frequency. Reporting throughout follows the ITRUSST consensus recommendations for standardized reporting of transcranial ultrasound parameters [84], and safety-relevant quantities are discussed against the companion biophysical safety consensus [16].

Terminology is used inconsistently in this literature, and several labels in common use denote overlapping rather than mutually exclusive categories. We therefore adopt the following conventions, applied consistently from this point onward. LIPUS (low-intensity pulsed ultrasound) denotes pulsed exposure at low intensity and is defined by intensity and pulsing, not by beam geometry. LIFU (low-intensity focused ultrasound; the variant “LIFUS” is not used further) denotes low-intensity exposure delivered through a focused transducer. FLIPUS (focused low-intensity pulsed ultrasound) is the intersection of the two and is simultaneously LIPUS and focused ultrasound. pFUS (pulsed focused ultrasound) denotes focused, pulsed exposure, usually above the LIPUS intensity range. FUS without qualification denotes focused exposure of unspecified intensity, and HIFU denotes focused exposure at ablative intensity. Focusing and intensity are therefore orthogonal axes rather than alternatives, and any classification that opposes “LIPUS” to “focused ultrasound” is a category error; Table 1 accordingly reports focusing and intensity in separate columns. Acoustic radiation force (ARF) is defined at first use in Section 1.2. Intracellular free calcium is written Ca^2+^ throughout, and sound pressure level is referenced to 20 µPa unless stated otherwise.

### 3.2. Low-Intensity Pulsed Ultrasound (LIPUS) and LIFU

Low-intensity focused ultrasound (LIFU) and LIPUS are applied at substantially lower intensities than ablative regimes in order to obtain predominantly non-thermal mechanical effects, principally ARF and, above the cavitation threshold, stable microbubble activity. The figures frequently quoted for this class—intensities below about 3 W/cm^2^ and focal temperature rises below about 0.5 °C—are not general physical boundaries and should not be presented as such. They are the operating values of particular neuromodulation protocols, and they sit alongside the regulatory ceiling for diagnostic imaging (derated ISPTA.3 ≤ 720 mW/cm^2^), which is a different quantity again. Actual heating scales with ISPTA, duty cycle, and exposure duration and is strongly amplified at bone interfaces and under reduced perfusion, so nominally low-intensity exposures can exceed 0.5 °C when applied for minutes or close to the skull [16,17]. These values are therefore reported here as protocol-specific, with the source and the metric for each given in Table 1 [16,17,83,84]. These low-intensity paradigms can reversibly modulate neural activity, likely via mechanical interactions with neuronal membranes and mechanosensitive or voltage-gated ion channels, rather than overt thermal injury [83]. Specifically, LIPUS activates integrin/FAK and PI3K/Akt pathways, enhances extracellular matrix production, angiogenesis, and cell survival, and accelerates bone, muscle, tendon–bone, and soft-tissue repair [86]. LIPUS also exerts robust immunomodulatory effects, decreasing pro-inflammatory cytokines and shifting macrophages from M1 to reparative M2 phenotypes via Wnt/β-catenin and related pathways [39]. It should be noted that the integrin/FAK, PI3K/Akt, and Wnt/β-catenin evidence summarized in this paragraph derives from musculoskeletal, macrophage, and bone-repair models rather than from neurons or glia; the corresponding neural evidence is considerably thinner and is set out in Section 5 and Section 6, with the model system for each study given in Table 1.

### 3.3. Focused Ultrasound (FUS) and High-Intensity Regimes (HIFU)

At the highest end of the energy spectrum, high-intensity focused ultrasound (HIFU) is typically used for thermal ablation, where acoustic intensities on the order of ~100–10,000 W/cm^2^ raise focal temperatures above ~55–60 °C (often up to ~60–85 °C), inducing protein denaturation, coagulative necrosis, and irreversible cell death [14]; this ablative regime is the only one considered in this review for which randomized, sham-controlled trials with prespecified clinical endpoints have been completed, in essential tremor and in Parkinson’s disease, and that evidence is set out in Section 8. Conversely, low-power FUS concentrates energy into millimeter-scale foci, enabling precise deep-brain neuromodulation and BBB opening. At neuromodulatory doses, FUS mechanically activates mechanosensitive ion channels and drives calcium influx and burst firing in cortical neurons without cavitation or heating. With intravenous microbubbles, low-power FUS induces stable cavitation and shear stress on cerebral vessels, causing transient, spatially confined blood–brain barrier opening that enhances delivery of chemotherapeutics, antibodies, and genes to brain tumors and neurodegenerative targets [87]. Two qualifications are required here. First, the exposures used for neuromodulation in rodents and in humans are not directly comparable: transcranial delivery in humans requires lower carrier frequencies, typically 200–650 kHz, to limit skull attenuation and aberration, at the cost of a larger focal volume, and the pressures quoted are usually free-water rather than in situ values [85,88]. Second, microbubble-mediated BBB opening is a mechanistically distinct regime defined by stable cavitation of an exogenous contrast agent rather than by direct mechanotransduction in the target cells; it was first demonstrated in rabbits at 1.63 MHz [89] and is now performed clinically at carrier frequencies in the low hundreds of kilohertz [46,47]. Conflating these three regimes—ablative HIFU, low-intensity neuromodulatory FUS, and microbubble-mediated BBB opening—is a frequent source of over-interpretation, and they are kept separate throughout this review and in Table 1.

### 3.4. Extracorporeal Shockwave Therapy (ESWT)

Extracorporeal shockwave therapy (ESWT) operates in yet another regime, delivering single, high-pressure shock pulses rather than continuous ultrasound. These impulses generate pronounced localized mechanical stresses and interstitial/extracellular responses that trigger neo-angiogenesis, vasodilation, recruitment of mesenchymal/stem cells, antifibrotic remodeling, and analgesia [90,91]. This ultimately supports regeneration in tendinopathy, bone, scars, and chronic wounds, leading to pain relief and enhanced vascularization in musculoskeletal disorders. ESWT dose is conventionally specified by energy flux density (EFD, in mJ/mm^2^) and by the number of impulses delivered per session, together with the interval between sessions; focused and radial devices differ substantially in pressure profile and rise time and are not interchangeable. Representative preclinical protocols relevant to neural and vascular outcomes use an EFD of 0.15 mJ/mm^2^ with 180 impulses per session on alternate days in a murine critical-limb-ischemia model [51], and three sessions of 1500 impulses at weekly intervals in a rat sciatic nerve crush model [52]. Both ESWT studies cited here report the number of impulses per session, the session interval, and the device used—0.15 mJ/mm^2^ at 180 impulses with a focused Duolith SD1 probe [51], and 1500 impulses per session with a focused Dornier MedTech device [52]. Energy flux density is reported in the first [51] but not in the second [52], where it is marked “NR” in Table 1 and has not been inferred from the device manufacturer. Because EFD and impulse number together determine the delivered dose, a protocol that specifies one without the other cannot be reproduced or compared quantitatively, and this is the respect in which the second study constrains synthesis.

### 3.5. Audible and Vibrotactile Stimulation

Audible and vibrotactile stimulation (20 Hz–20 kHz) can act systemically through somatosensory and auditory pathways. Rhythmic sensory or vibrotactile stimulation near 40 Hz entrains cortical gamma activity and, in mouse models of amyloid and tau pathology, is associated with outcomes that differ by modality and by model and should be attributed separately: reduced amyloid load in the visual cortex after visual stimulation; reduced amyloid in the auditory cortex and hippocampus, together with reduced phosphorylated tau in a tauopathy model and improved spatial and recognition memory, after auditory stimulation; a further reduction in the medial prefrontal cortex obtained only with combined auditory and visual stimulation and with neither modality alone; and reduced DNA damage, preserved synaptic proteins, and improved motor performance after whole-body vibrotactile stimulation [33,36,37]. At the cellular level, audible and low-kHz stimuli can reorganize the cytoskeleton and chromatin, alter mechanosensitive gene transcription, and modulate inflammatory signaling in a cell-type- and frequency-dependent manner [39]. Three physically distinct exposures are routinely grouped under the label “40 Hz”, and they should not be conflated. (i) Sensory 40 Hz stimulation, delivered as light flicker or as 40 Hz tone trains, acts through the retina or the cochlea; the resulting gamma entrainment is a network-level phenomenon driven by afferent input, not a direct mechanical effect on the cells in which the downstream pathology is measured [36,37]. (ii) Vibrotactile or whole-body 40 Hz stimulation delivers mechanical displacement to the body surface—approximately 16 µm peak displacement for 1 h per day over three to six weeks in the published tauopathy and CK-p25 protocols [33], or 0.8 mm vertical amplitude twice daily for two months in aged rats—and reaches the brain through somatosensory afferents, through cortical entrainment, and potentially through direct tissue vibration; these routes have not been dissociated experimentally in any of the cited studies. In both protocols, the unstimulated group was housed on unpowered or non-vibrating plates, so that both groups were exposed to comparable background sound [33,34]. (iii) A 40 Hz pulse-repetition frequency imposed on a megahertz ultrasound carrier is not a 40 Hz mechanical oscillation at all: the tissue experiences 2 MHz pressure cycles delivered in 400 µs bursts at ISPPA 190 W/cm^2^ and ISPTA 3.0 W/cm^2^, and the figure of 40 Hz describes only the envelope of the pulse train [43]. Any claim of an optimal 40 Hz band is consequently restricted, throughout this review, to the specific exposure type, model, and outcome in which it was tested and is not generalized across these three categories.

### 3.6. Separating Sensory-Pathway Activation, Network Entrainment, and Direct Cellular Effects

A substantial part of the evidence attributed to acoustic mechanotransduction in vivo is equally compatible with activation of the auditory pathway, and the two can be separated only with specific controls. The two studies most often cited together on this point used different species, different readouts, and different manipulations, and their results should not be merged. In guinea pigs, transcranial ultrasound evoked extensive activation of the primary auditory and primary somatosensory cortex across a wide range of exposure parameters, including single pulses and pulse-repetition frequencies as low as 10 Hz, and bilateral transection of the auditory nerves or bilateral removal of cochlear fluids eliminated that activation, localizing the route to the cochlea and the auditory nerve; no cochlear ablation was performed in that study. In mice, wide-field calcium imaging showed a cortical activation pattern closely resembling the response to audible sound and unlike the response to light flashes, with the auditory cortex recruited earliest and at the lowest intensities and with a contralateral bias consistent with an inner-ear route; there, chemical deafening with kanamycin and furosemide reduced electromyographic motor responses to both ultrasound and audible sound while leaving air-puff responses intact, and uncoupling the transducer from the head abolished the motor response, but the authors state explicitly that chemical deafening could not be carried out in the older transgenic animals used for imaging because of high mortality, so no cortical data from deafened animals exist and the deafening result in that study is a motor result and not a cortical one [48,49]; in humans, transcranial ultrasound is accompanied by an audible confound that can be effectively masked, and unmasked protocols cannot distinguish direct neuromodulation from auditory startle or arousal [92]. The counter-evidence is equally specific and must be given its weight: in wild-type normal-hearing mice and in two strains of genetically deaf mice, elimination of peripheral auditory pathway activation did not abolish motor responses to ultrasound, and smoothing the sharp edges of a rectangular stimulus envelope abolished the auditory brainstem response while leaving the motor response intact, which dissociates the two at the level of the stimulus waveform [50]; and, in a mouse preparation designed to minimize the confound, responses persisted under conditions controlling for auditory activation [93]. The methodological conclusion is not that in vivo ultrasound neuromodulation is an artifact but that a claim of direct in vivo neuromodulation now requires, at minimum, a sham transducer matched for audible output, a deafened or acoustically masked control group, and preferably a smooth-envelope waveform that reduces audible transients. Table 1 records, for every in vivo entry, whether such a control was reported.

The same distinction applies to the audible-sound and music literature, and it changes how that literature should be read. Increases in hippocampal and cortical BDNF after music exposure or acoustically enriched housing are the output of an intact auditory system together with the arousal, attention, and general environmental-enrichment components of those paradigms; they document experience-dependent plasticity, and they are not evidence that acoustic pressure directly gates neuronal mechanosensors. None of the enrichment studies cited in Section 5 included a deafened or masked control, and none applied a quantified mechanical dose to the tissue of interest. By contrast, the in vitro audible-sound work in which a defined sound pressure level is applied directly to cultured cells does constitute direct mechanical stimulation, but it has so far been performed almost exclusively in non-neural lines [31,32]. Three levels of claim are therefore kept distinct throughout this review: sensory-pathway activation, network-level entrainment, and direct cellular mechanotransduction. Where a study cannot discriminate between them, that is stated rather than resolved in favor of the mechanistically more interesting option.

**Figure 2:**
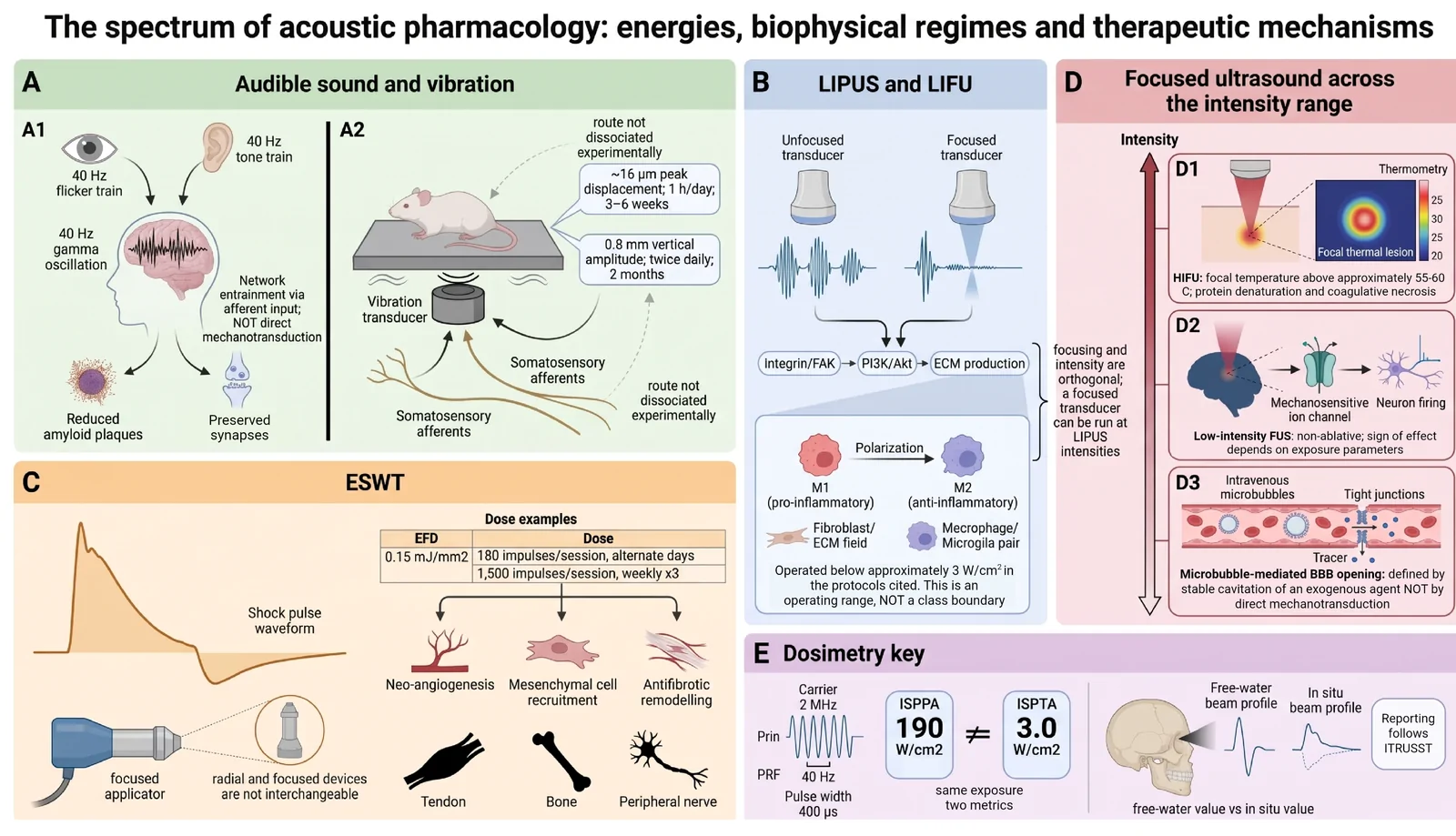
The spectrum of acoustic pharmacology: energies, biophysical regimes, and therapeutic mechanisms. (**A**) Audible sound and vibration, shown as two physically distinct exposures that are frequently conflated. (A1) Sensory 40 Hz stimulation (light flicker or tone trains) acts through the retina or the cochlea; the resulting cortical gamma entrainment is a network phenomenon driven by afferent input and is associated, in mouse amyloid and tau models, with reduced amyloid load and preserved synapses [36,37]. (A2) Vibrotactile and whole-body 40 Hz vibration delivers mechanical displacement to the body surface (≈16 µm peak displacement, 1 h/day for 3–6 weeks [33], against a no-vibration control matched for background acoustic noise; 0.8 mm vertical amplitude twice daily for 2 months [34]) and reaches the brain through somatosensory afferents and, potentially, direct tissue vibration; these routes have not been dissociated experimentally. (**B**) LIPUS and LIFU. Pulsed low-intensity exposure, operated below approximately 3 W/cm^2^ in the protocols cited, delivers non-thermal mechanical cues that engage integrin/FAK and PI3K/Akt signaling, enhance extracellular matrix (ECM) production, and shift microglial and macrophage polarization from a pro-inflammatory M1 state toward a reparative M2 phenotype [39,83,86]. The 3 W/cm^2^ figure is the operating range of these protocols and not a physical boundary between classes. (**C**) Extracorporeal shockwave therapy (ESWT). Single high-pressure shock pulses, dosed as energy flux density (EFD, mJ/mm^2^) and impulses per session—for example, 0.15 mJ/mm^2^ with 180 impulses per session [51], or 1500 impulses per session at weekly intervals [52]—generate localized mechanical stress that triggers neo-angiogenesis, mesenchymal cell recruitment, and antifibrotic remodeling, principally in musculoskeletal and vascular targets [90,91]. (**D**) Focused ultrasound across the intensity range, shown as three regimes that are mechanistically distinct. (D1) HIFU: thermal ablation at focal temperatures above approximately 55–60 °C, producing protein denaturation and coagulative necrosis [14]. (D2) Low-intensity FUS: non-ablative deep-brain neuromodulation through gating of mechanosensitive channels [30,83], with the sign of the effect depending on exposure parameters [94]. (D3) Microbubble-mediated BBB opening: a separate regime defined by stable cavitation of an exogenous contrast agent rather than by direct mechanotransduction in the target cells [13,87,89]. (**E**) Dosimetry key. The same exposure yields very different figures depending on the metric reported: for a 2 MHz carrier pulsed at 40 Hz with 400 µs pulses, an ISPPA of 190 W/cm^2^ corresponds to an ISPTA of 3.0 W/cm^2^ [43]; free-water and in situ values differ because the skull attenuates, scatters, and aberrates the beam in a frequency-dependent manner [85]. Reporting throughout follows the ITRUSST standard [16,84]. Evidence and dosimetry note: all numerical values shown are those reported by the cited primary sources and are reproduced in full, with their original metrics and with free-water versus in situ annotation, in Table 1. Panels (**B**) and (D2) overlap deliberately because focusing and intensity are orthogonal parameters and a focused transducer can be operated at LIPUS intensities (Section 3.1). Panel (**A**) depicts exposures that act, at least in part, through afferent pathways rather than through direct mechanotransduction in the cells in which the outcome is measured (Section 3.6). Figure concepts were derived from refs [7,14,16,30,33,34,36,37,42,43,51,52,83,84,85,90,91]. Created with Created in Biorender. Giovanni Luca Cipriano. (2026) https://BioRender.com/jk5wjt9.

The translation of these physical principles—spanning acoustic radiation force, fluid shear stress, and cavitation dynamics—into distinct non-thermal therapeutic regimes dictates the cellular response boundary, as graphically summarized in Figure 2.

## 4. Comparative Analysis of Acoustic Energies

The biological efficacy of acoustic stimulation is strongly influenced by the architecture of the waveform, which shapes how cells interpret mechanical cues through mechanotransduction pathways [32]. In murine ST2 bone-marrow stromal cells, exposure to a pure 440 Hz sine wave at 94 dB SPL—which, referenced to the conventional airborne value of 20 µPa, corresponds to approximately 1 Pa—downregulates mechanosensitive genes such as Ptgs2, Ctgf, and Tnc, with mRNA levels reduced to roughly 60–80% of baseline within 1–2 h [31]. This suppression persists for several hours after the stimulus ends, indicating that a coherent sinusoidal waveform can act as a stable mechanical signal that durably modulates mechanosensitive gene transcription in these cells [31]. The effect has not been reproduced in neurons or glia, and its generalization to neural cells remains untested.

When the same cells are stimulated with 440 Hz triangle or square waves at identical sound pressure levels, the suppressive effect on these genes is markedly attenuated or absent, demonstrating that waveform, rather than frequency per se, is a key determinant of gene regulation [28,31]. Square waves inherently contain a series of high-frequency harmonics, and transcriptomic comparison shows that the gene-expression pattern induced by a 440 Hz square wave more closely resembles that produced by higher-frequency stimulation (for example, 14 kHz) than that produced by a pure 440 Hz sine wave [31,32].

These findings indicate that waveform purity and spectral content shape the cellular response: sinusoidal profiles yield more reproducible mechanotransductive effects, whereas non-sinusoidal waveforms introduce additional spectral components that shift the biological outcome toward a high-frequency-like response [32]. This conclusion currently rests on work from a single laboratory in non-neural cell lines, and independent replication—particularly in neurons and glia—is a priority.

Therapeutic effects of acoustic and vibrational stimulation show a pronounced frequency dependence. An optimal band near 40 Hz (the gamma range) has been reported for neural modulation and regeneration but only across a small and heterogeneous set of preclinical paradigms: vibrotactile stimulation in mouse tauopathy and CK-p25 models [33], whole-body vibration in aged rats [34], and low-magnitude vibration of SH-SY5Y cells in vitro [35]. Higher-frequency regimens have been linked to dysfunction or stress only in specific experimental contexts, so frequency-dependent effects are not uniformly generalizable.

Whole-body or transcranial 40 Hz vibration entrains cortical gamma activity, increases c-Fos in the somatosensory and motor cortex, and improves cognition or motor performance in models of aging or neurodegeneration [95]. In Tau P301S and CK-p25 mice, weeks of daily 40 Hz vibrotactile stimulation reduce phosphorylated tau, DNA damage, and neuronal loss and improve motor function [33]. In aged rats, eight weeks of 40 Hz whole-body vibration elicit gamma oscillations, enhance hippocampal astrocyte–neuron synaptic function, reduce neuronal apoptosis, and improve cognition [34]. In none of these studies was the frequency titrated systematically against closely spaced neighboring values, and in none was the mechanical route separated from afferent sensory drive; the apparent convergence on 40 Hz should therefore be read as a hypothesis generated across heterogeneous paradigms rather than as an established optimum. The 40 Hz pulse-repetition frequency used in transcranial ultrasound studies [43] is a physically different exposure (Section 3.5) and does not constitute independent support for this band.

At the cellular level, 40 Hz vibration enhances differentiation and neurite elongation in neuron-like SH-SY5Y cells more effectively than 100 Hz, supporting an optimal window for growth and maturation in that cell line [35]. Multiple forms of physical stimulation, including electrical fields, drive neurite outgrowth in PC12 cells through p38 MAPK activation, which is necessary and sufficient for neuronal differentiation [96]; low-frequency mechanical stimulation near 40 Hz is consistent with engagement of similar MAPK pathways in neuronal models, although this has not been tested directly [97].

In non-neural cells, low-frequency sound around 100 Hz enhances fibroblast migration, whereas higher frequencies (200–1600 Hz) show weaker or different effects, indicating a frequency-dependent window for orderly motility and actin remodeling [98]. Very high acoustic frequencies (ultrasound, MHz) can be pro-regenerative but require careful dose control: low-intensity ultrasound (1–3 MHz) improves peripheral nerve regeneration, whereas higher-dose regimens begin to impair survival or differentiation of neural stem cells [99]. Biophysical analyses of ultrasound neuromodulation show that as carrier frequency increases, reduced membrane capacitance and altered coupling demand higher pressures to reach neuronal excitation, narrowing the safe therapeutic window [97]. High-frequency ultrasound can also produce spike-frequency-dependent inhibition as well as excitation, so the sign of the effect is not fixed by the energy class alone [94].

Finally, the composition of the literature itself constrains what can be concluded from it. Of the 144 sources cited in this review, 59 (41.0%) are reviews, consensus statements, or guidelines, 37 (25.7%) report animal in vivo experiments, 27 (18.8%) report in vitro work, 14 (9.7%) are biophysical or computational analyses, 5 (3.5%) are human feasibility, safety, or observational studies, and 2 (1.4%) are randomized controlled trials with a clinical endpoint, both of ablative focused ultrasound (Table 2). Grouped by acoustic energy (Table 3), 36 sources (25.0%) concern focused ultrasound including ablative high-intensity focused ultrasound and microbubble-mediated BBB opening, 21 (14.6%) concern audible sound or vibration, 19 (13.2%) concern LIPUS or LIFU, 6 (4.2%) concern sonogenetics, and 5 (3.5%) concern shockwaves, while 57 (39.6%) supply non-acoustic mechanobiological or molecular background rather than acoustic data. Two features of this distribution bear directly on the argument of this review. First, non-acoustic background mechanobiology is the largest single block, which is precisely why the membrane-to-chromatin axis of Section 2 must be presented as a hypothesis assembled from non-acoustic sources rather than as an established pathway. Second, the human evidence is small and unevenly distributed. Five of the seven human studies are feasibility, safety, or observational reports, and only one characterizes the auditory confound [45,46,47,92,100]. The two randomized, sham-controlled trials with a clinical endpoint both concern ablative focused ultrasound (Section 8), so they bear on thermal lesioning and not on the non-ablative regimes whose mechanisms this review sets out; for those regimes, the human evidence remains confined to safety and feasibility.

## 5. Acoustic Neurotrophy and Regenerative Signaling

Mechanical stimulation of neural architectures, such as ultrasound applied to injured dorsal root ganglion (DRG) neurons, activates mTOR-dependent growth programs that support cellular repair and survival. In axotomized DRG neurons, ultrasound stimulation significantly increases neurite number and length, concomitant with enhanced mTOR activity and upregulation of the regeneration-associated protein GAP-43, indicating that mTOR pathway activation contributes to neurite regeneration and peripheral nerve repair [38]. The exposure used in that study was a 1.5 MHz carrier pulsed at 10 Hz with a 20% duty cycle for 10 min, applied once to rat DRG explants immediately after axotomy; the 100 Hz pulse-repetition condition was less effective, and no functional recovery endpoint was assessed (Table 1).

Parallel to high-frequency ultrasound, low-frequency mechanical vibrations can also activate kinase-mediated signaling pathways associated with neurotrophic responses. Mechanical vibration and related physical stimuli have been shown to engage MAPK cascades and other kinase networks in mechanically loaded non-neural tissues, linking mechanical cues to changes in survival, inflammation, and differentiation [101].

Vibrations at 40 Hz also enhance neurite elongation and differentiation in SH-SY5Y neuron-like cells, further supporting a robust pro-neuritogenic effect of low-frequency mechanical stimulation [35]. This observation was made in an immortalized human neuroblastoma line exposed to low-magnitude vibration (<1 g), with vibration at 100 Hz as the only comparator, so it establishes a frequency preference within that model rather than an optimum for neurons generally.

**Figure 3:**
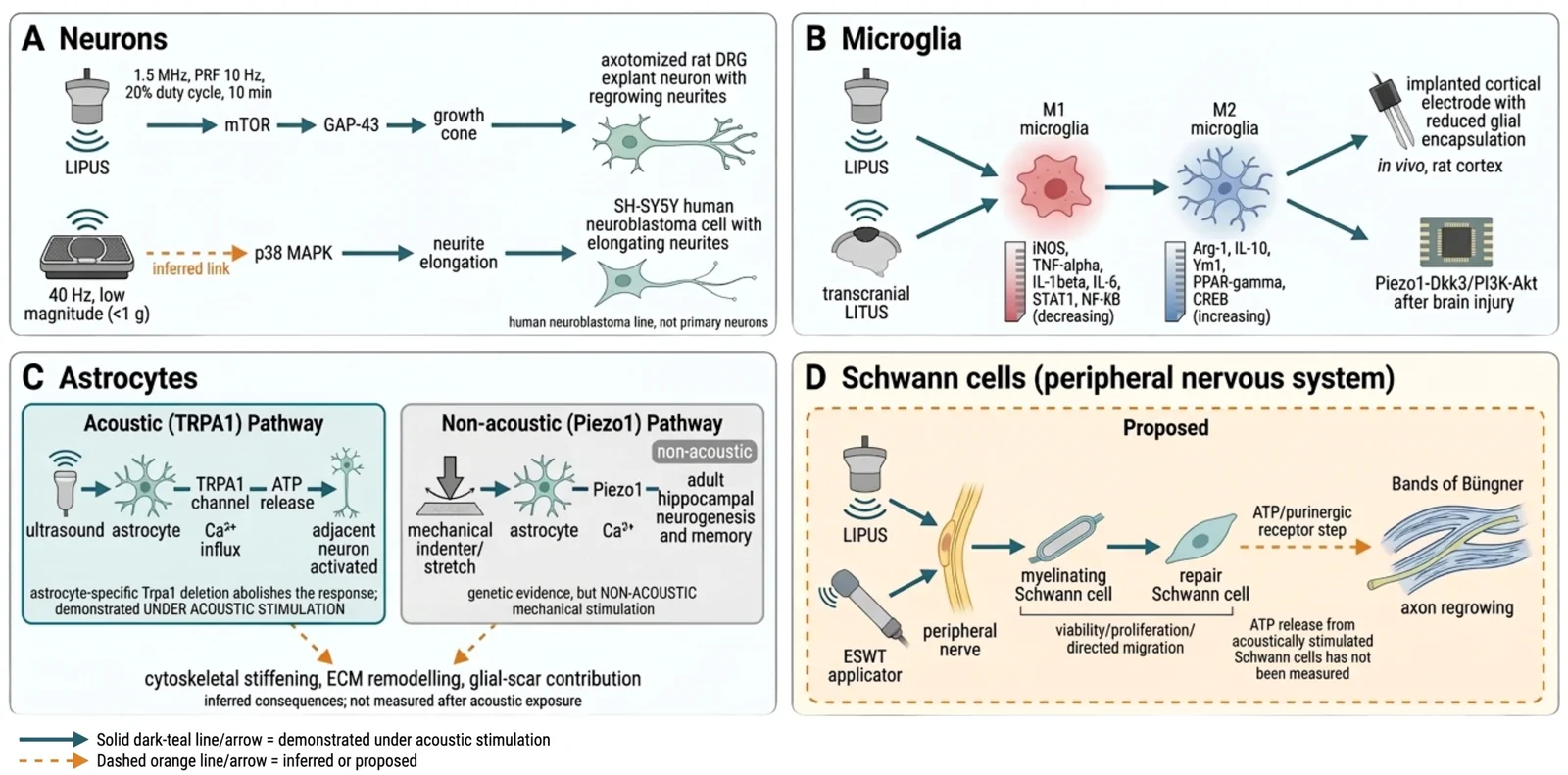
Acoustic modulation of neuronal and glial phenotypes, with the level of evidence indicated for each cell type. (**A**) Neurons. Low-intensity pulsed ultrasound (1.5 MHz carrier, 10 Hz pulse-repetition frequency, 20% duty cycle, 10 min) increases neurite number and length in axotomized rat dorsal root ganglion explants, with mTOR activation and GAP-43 upregulation [38]; 40 Hz low-magnitude vibration increases neurite elongation and differentiation in the SH-SY5Y human neuroblastoma line more effectively than 100 Hz [35]. p38 MAPK is drawn as an inferred rather than a demonstrated link between low-frequency vibration and neurite outgrowth. (**B**) Microglia. LIPUS shifts LPS-primed microglia from an M1 toward an M2-like phenotype, reducing iNOS, TNF-α, and STAT1 and increasing Arg-1, IL-10, and PPARγ [40], suppresses NF-κB while restoring CREB signaling [121], and reduces microglial activation in vivo around implanted cortical electrodes [41]; low-intensity transcranial ultrasound engages a Piezo1–Dkk3/PI3K–Akt pathway after brain injury [122]. (**C**) Astrocytes. Two mechanosensors are shown and are distinguished by the evidence available for each: astrocytic TRPA1, which is required for the Ca^2+^ response to low-intensity, low-frequency ultrasound and for the downstream neuronal activation, demonstrated by cell-type-specific gene deletion under acoustic stimulation [23]; and astrocytic Piezo1, which determines adult hippocampal neurogenesis and hippocampus-dependent memory, demonstrated genetically but with non-acoustic mechanical stimulation [118]. The downstream cytoskeletal stiffening and ECM remodeling shown are consequences inferred from these pathways rather than measured after acoustic exposure. (**D**) Schwann cells (proposed). LIPUS enhances Schwann cell viability, proliferation, and directed migration and is associated with a repair-phenotype transition and the formation of bands of Büngner [127,128]; the ATP/purinergic step drawn here is a proposal because ATP release from acoustically stimulated Schwann cells has not been directly measured [126]. Evidence key: solid outlines and solid arrows mark relationships demonstrated under acoustic stimulation; dashed outlines and dashed arrows mark inferred or proposed relationships, namely p38 MAPK in panel A, the cytoskeletal and ECM consequences in panel C, and ATP release in panel D. Figure concepts were derived from refs [23,35,38,40,41,118,121,122,126,127,128]. Created with Created in Biorender. Giovanni Luca Cipriano. (2026) https://BioRender.com.

A p38 mitogen-activated protein kinase (MAPK)–CREB cascade is the mediator most often proposed for this vibration-induced response, and it is a plausible candidate because that cascade regulates cellular stress responses, differentiation, and cytoskeletal remodeling in neuronal and other mechanosensitive cell types [101,102]. It is, however, a hypothesis rather than a demonstrated mechanism, and it is designated as such here and in Figure 3A. The evidence that supports it was obtained in other systems: p38 MAPK is necessary and sufficient for neurite outgrowth in electrically stimulated PC12 cells [96], and p38- and ERK-dependent mechanotransduction has been documented in mechanically loaded bone and mesenchymal models [101,103]. Neither an electrical stimulus in a pheochromocytoma line nor mechanical loading of non-neural tissue establishes the mechanism of action of 40 Hz vibration on neuronal cells, and to the best of our knowledge, no study has tested p38 MAPK or CREB involvement in neurons exposed to low-frequency vibration. If this cascade is operative, mechanical stimuli would convert physical energy into biochemical signals accompanying neuronal growth, neurite outgrowth, and structural repair in vitro; on present evidence, that step is inferred from the systems in which each element was measured separately and not from any single neuronal preparation [102,103].

The biochemical activation of mechanically sensitive kinase pathways is subsequently translated into measurable structural regeneration. Ultrasound and other mechanical cues activate pathways such as mTOR and MAPKs, which in turn regulate local protein synthesis and cytoskeletal remodeling required for axon extension [38,104].

One of the most widely recognized markers of axonal repair is growth-associated protein 43 (GAP-43), a neuron-specific protein critically involved in axonal growth, guidance, and synaptic remodeling during development and after injury [105]. GAP-43 levels and phosphorylation state tightly track regenerative growth in many CNS and PNS models, and axonal GAP-43 synthesis supports elongating axon growth [106].

Experimental evidence indicates that targeted ultrasound stimulation can significantly increase intracellular GAP-43 expression in injured dorsal root ganglion neurons, in parallel with activation of the mTOR pathway, a key regulator of regenerative axon outgrowth [38]. GAP-43 acts downstream of mTOR, and its upregulation is associated with enhanced axonal sprouting and outgrowth in regenerating axons [38].

As a consequence of this localized protein synthesis, neurons exposed to acoustic stimulation frequently display pronounced neurite extension and increased total neurite length compared with unstimulated controls, indicating active structural remodeling of neural architecture [38,104]. These GAP-43-linked changes are consistent with broader work indicating that GAP-43 contributes to axonal plasticity, although recent analyses caution that its expression alone should not be regarded as generally sufficient to drive regeneration across neuronal populations [106].

Beyond local mechanotransduction, acoustic stimulation is linked to systems-level BDNF/TrkB signaling that supports hippocampal plasticity and regeneration.

Environmental acoustic enrichment and music exposure increase BDNF expression in the hippocampus and other auditory-related regions in rodents and chicks [107,108,109]. Music or acoustically enriched housing elevates BDNF and TrkB in the hippocampus and prefrontal cortex, alongside increased dendritic spine density and synaptic proteins (SYP, PSD95), indicating enhanced structural plasticity [107]. Enriched environments more broadly upregulate hippocampal BDNF via epigenetic promoter modifications and support neurogenesis [110]. In humans, music-based interventions and training also modulate peripheral and central neurotrophic factors, including BDNF, consistent with molecular neuroplasticity effects [111]. These paradigms act through an intact auditory system and carry substantial arousal, attention, and general environmental-enrichment components; they therefore document experience-dependent plasticity rather than direct mechanical activation of neuronal mechanosensors. None of the studies cited in this paragraph included a deafened or acoustically masked control group or quantified a mechanical dose delivered to the hippocampus, which are the controls that would be required to separate sensory-pathway activation from direct cellular mechanotransduction [48,49,92,93] (Section 3.6).

BDNF exerts its effects mainly through TrkB. Binding of BDNF to TrkB induces receptor dimerization and autophosphorylation, creating docking sites that recruit multiple intracellular cascades [112]. Acoustic enrichment and sound stimulation increase mature BDNF protein in the auditory brainstem and hippocampus, and BDNF is required for activity-dependent refinement of auditory circuits [113,114].

After TrkB activation, principal downstream pathways include PI3K/Akt and MAPK/ERK, as well as PLCγ/PKC [107]. Music-enriched environments upregulate PI3K, Akt, ERK/MAPK, and PLCγ1/PKC at mRNA and protein levels in the hippocampus and prefrontal cortex, correlating with increased dendrites and spine density [107]. These cascades regulate neuronal survival, synaptic plasticity, and metabolic adaptation and are central to BDNF-mediated neurogenesis and long-term potentiation [115].

Through these pathways, acoustic enrichment (including music and structured sound) improves hippocampal LTP, learning, and memory and can partially reverse noise- or stress-induced auditory cortical dysfunction, with recovery of BDNF expression [109]. An optimized shockwave stimulation protocol has similarly been shown to increase hippocampal neurogenesis via neurotrophic signaling, without a significant improvement in spatial memory [53], suggesting that these principles may extend to other forms of mechanically delivered acoustic energy, pending direct confirmation.

## 6. The Glial Mechanome

Glial cells are now recognized as active mechanosensitive regulators of neural tissue rather than passive metabolic support. Glia detect changes in extracellular stiffness, strain, and fluid forces via integrin-based adhesions and stretch-activated ion channels, converting mechanical cues into biochemical signals that drive remodeling of the neural microenvironment [116]. Through this mechanotransduction machinery, glial cells dynamically adjust both the biochemical milieu and the physical properties of nervous tissue in health and disease [117].

Astrocytes are central regulators of CNS mechanical properties and rely on mechanosensitive channels to interpret local physical cues [116,117]. The Piezo1 channel is highly expressed in astrocytes and acts as a key mechanoreceptor: its activation by mechanical stimuli induces Ca^2+^ influx, triggering downstream signaling and ATP release that supports adult neurogenesis and cognitive function [118]. This attribution is genetic rather than correlative: astrocyte-specific deletion of Piezo1 impairs, and astrocytic overexpression enhances, adult hippocampal neurogenesis and hippocampus-dependent memory in mice [118]. The corresponding evidence for Piezo1 in microglia is distinct and is reviewed separately [119]. Piezo1-mediated Ca^2+^ signaling is coupled to cytoskeletal remodeling, enabling astrocytes to adjust their stiffness, morphology, and interactions with the extracellular matrix (ECM) in response to mechanical changes [116,117]. These structural adaptations shape ECM composition and contribute to glial scar formation, which constitutes both a mechanical and biochemical barrier after CNS injury [41]. Beyond their mechanosensitivity, astrocytes maintain synaptic function and fluid homeostasis and modulate neuroinflammation within the healthy and diseased brain [117].

Microglia are highly dynamic immune sentinels of the brain parenchyma, capable of rapidly altering morphology and phenotype in response to environmental signals [120]. Low-intensity pulsed ultrasound (LIPUS) can modulate microglial activation, shifting cells away from a pro-inflammatory M1-like state toward an anti-inflammatory, reparative M2-like phenotype. In LPS-primed microglia, LIPUS reduces expression of M1 markers such as iNOS, TNF-α, IL-1β, and IL-6, while enhancing M2-associated mediators including Arg-1, IL-10, and Ym1 [40]. This polarization shift is mediated in part by STAT1/STAT6/PPARγ signaling and accompanies decreased NF-κB activation [121]. More broadly, ultrasound can suppress pro-inflammatory cascades by inhibiting TLR4/MyD88/NF-κB signaling and c-Jun N-terminal kinase, thereby lowering production of cytotoxic cytokines [40,121]. Low-intensity transcranial ultrasound stimulation (LITUS) further attenuates neuroinflammation and brain injury by engaging a Piezo1-Dkk3/PI3K-Akt pathway, leading to reduced IL-1β, IL-6, and TNF-α levels and improved functional outcomes after cardiac arrest-related brain injury [122]. These findings align with broader evidence that Piezo1 and other mechanosensitive channels shape microglial responses to mechanical changes in the CNS [41,119,120].

Myelinating glia critically regulate the speed and timing of action potential propagation. In the CNS, oligodendrocytes and their progenitors (OPCs) continuously monitor neuronal activity and adjust myelin formation accordingly [123]. Neuronal firing and action potential propagation can promote ATP release into the extracellular space, where purinergic signaling acts as a key regulator of OPC proliferation, differentiation, and myelination [124]. ATP and its metabolites (ADP, adenosine) act on P2 and P1 receptors expressed by OPCs and oligodendrocytes to influence lineage progression and myelin sheath formation, thereby coupling neuronal activity to the structural refinement of neural circuits [123]. Through these mechanisms, bioelectric and associated mechanical changes in active axons converge with purinergic signaling to optimize myelination and circuit timing [125].

In the peripheral nervous system, Schwann cells display remarkable plasticity during nerve regeneration. After injury, mature Schwann cells undergo an EMT-like reprogramming into repair Schwann cells, which clear myelin debris, form bands of Büngner, secrete regeneration-promoting factors, and organize a permissive scaffold that guides regrowing axons [126]. Physical and mechanical stimulation can influence Schwann cell migration, differentiation, and alignment, thereby modulating peripheral nerve repair. Low-intensity pulsed ultrasound has been reported to enhance Schwann cell viability, proliferation, and directed migration and to increase the secretion of neurotrophic factors that support axonal regrowth in peripheral nerve injury models [127]. ATP-mediated purinergic signaling is a major mechanotransduction pathway in CNS glia, and Schwann cells also engage ATP-dependent communication during regeneration [117,127], although the specific pathways by which acoustic stimulation induces ATP release from Schwann cells warrant further investigation in future studies.

By coupling acoustic neurotrophy with dynamic glial reprogramming, mechanical stimulation orchestrates a synergistic regenerative microenvironment, as detailed in Figure 3.

## 7. Translational Frontiers

In the context of Alzheimer’s disease (AD), mechanical stimulation through focused ultrasound (FUS) has been investigated as a strategy for reducing pathological burden and promoting clearance of amyloid aggregates through two complementary mechanisms, with evidence at present confined to rodent models and to small open-label human safety series. Repeated FUS with microbubbles (FUS-MB) opens the blood–brain barrier (BBB), recruits and activates microglia and other immune cells, and lowers Aβ plaque and phosphorylated tau burden while improving memory [45]. Following sonication, microglia accumulate around plaques and actively engulf Aβ deposits. Similarly, transcranial focused ultrasound (tFUS) delivered with a 2 MHz carrier at a 40 Hz pulse-repetition frequency has been shown to stimulate microglial activity and reduce Aβ burden by approximately 50% in 5xFAD mouse models [43].

In parallel, FUS-MB promotes the removal of soluble Aβ by increasing its drainage from brain tissue into the cerebrospinal fluid (CSF) along glymphatic and lymphatic pathways [44], enhancing biological clearance rather than causing direct physical fragmentation of existing plaques [45]. Early clinical studies show that FUS-MB can safely and reversibly open the BBB in patients with Alzheimer’s disease: the first trial treated five patients in a two-stage open-label design [46], and a subsequent series achieved reversible hippocampal opening of 14–71% of hippocampal volume across 17 treatments in six patients [47]. These are phase I feasibility and safety studies. They were not designed to demonstrate clinical efficacy and do not do so; reported reductions in amyloid PET signal are small and uncontrolled, and the suggestion that BBB opening might potentiate anti-amyloid antibodies remains a hypothesis under investigation rather than a demonstrated effect [46,47,129].

In neuropathies and peripheral nerve injuries, acoustic stimulation has been explored as a strategy to support nerve repair and functional recovery. Low-intensity pulsed ultrasound (LIPUS) stimulates Schwann cell proliferation and migration while promoting their transition toward regenerative repair phenotypes. These effects are accompanied by increased expression of neurotrophic factors such as nerve growth factor (NGF) and brain-derived neurotrophic factor (BDNF), together with activation of pro-survival signaling cascades including PI3K/Akt and TrkB/Akt/CREB [128]. In experimental models of diabetic neuropathy, LIPUS delivered at 1 MHz improves pain-related behavior, preserves myelin morphology, and increases axonal markers. These functional and structural improvements are associated with reduced malondialdehyde (MDA) levels and changes in Keap1/Nrf2/HO-1 signaling [42]. Overall, current reviews describe LIPUS as a promising adjunct therapy capable of prolonging the Schwann cell repair state and enhancing peripheral nerve regeneration [130].

Extracorporeal shockwave therapy (ESWT) enhanced perfusion and angiogenic responses in a mouse model of critical limb ischemia [51]. Separately, ESWT significantly improved gait parameters after sciatic nerve crush in rats, while the accompanying increase in myelination and myelin basic protein was a non-significant trend [52]; the direct contribution of vascular mechanisms to these neural outcomes requires further investigation. Although the molecular mechanisms are less extensively characterized in the specific studies cited, ESWT is frequently discussed together with LIPUS in regenerative medicine overviews as a complementary modality capable of facilitating nerve repair through improved vascular support and tissue remodeling [91,131,132,133].

The advent of sonogenetics represents a paradigm shift toward wireless, non-invasive control of neural circuits by combining ultrasound with genetically encoded mediators [56,82,134]. This approach operates by engineering specific target cells to express ultrasound-sensitive proteins, enabling cell-type-specific activation in deep brain regions [134,135]. In doing so, sonogenetics may help address the depth-penetration limits of optogenetics and the cell-specificity challenges of purely physical ultrasound or other non-invasive approaches [136].

By exploiting acoustic radiation force and related mechanical effects, ultrasound can deform neuronal membranes and increase membrane tension, which is then converted into depolarization via mechanosensitive ion channels [137,138].

At the molecular forefront are engineered bacterial mechanosensitive channels, especially the large-conductance MscL mutants such as MscL-G22S and MscL-I92L, which show markedly enhanced ultrasound sensitivity [55,136]. When expressed in mammalian hippocampal or striatal networks, these MscL variants allow low-pressure, focused ultrasound to reliably drive action potentials, amplify evoked potentials, and sculpt network oscillations or behavior with sub-second precision [55,135,136].

In parallel, genetic introduction of transient receptor potential (TRP) channels (e.g., TRPV1 in sonothermogenetics and TRP-4 in the original C. elegans work) confers ultrasound responsiveness to defined neural subsets [15,54,139]. The two components of these approaches must, however, be separated when their invasiveness is described. The ultrasound exposure itself is non-invasive, whereas delivering the actuator requires administration of a viral vector—in current rodent work by intracranial injection or by high-dose systemic adeno-associated virus—with the attendant requirements for surgical access or large vector doses, promoter-dependent cell-type specificity, immunogenicity, and stable long-term transgene expression [140]. Sonogenetics is therefore more accurately described as a minimally invasive stimulation modality built upon an invasive and currently irreversible genetic intervention, and the descriptor “non-invasive” applies to the stimulation step alone. A further distinction applies to TRPV1-based sonothermogenetics, which operates through a focal temperature rise measured by magnetic resonance thermometry rather than through membrane strain [15]; it therefore does not inherit the thermal-safety profile of low-intensity mechanical neuromodulation and must be evaluated against thermal-dose criteria [16].

**Figure 4:**
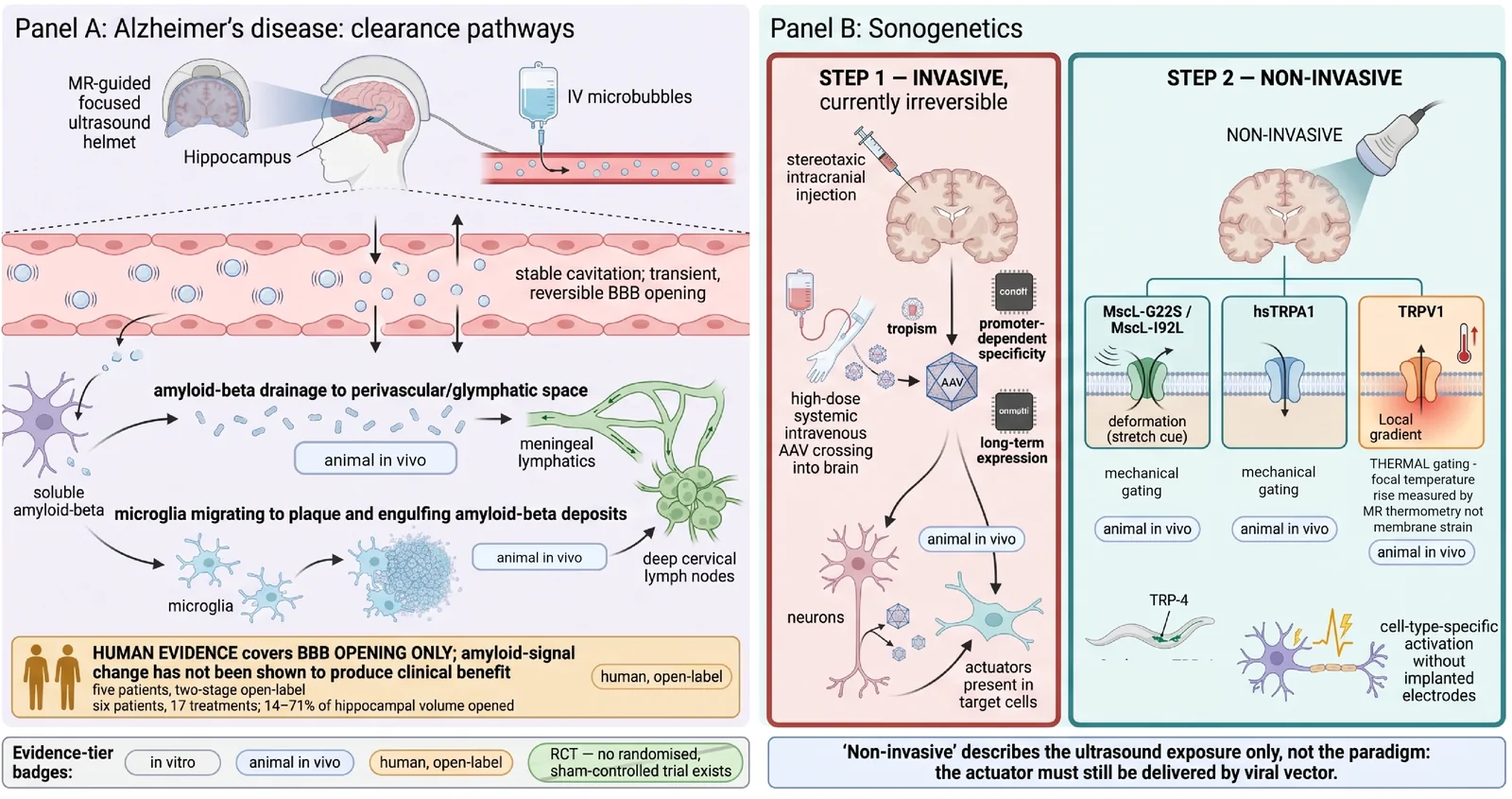
Translational frontiers of acoustic neuromodulation, annotated by level of evidence. (**A**) Alzheimer’s disease clearance pathways. Focused ultrasound with intravenously administered microbubbles (FUS-MB) produces stable cavitation and shear stress that transiently open the blood–brain barrier (BBB). Two clearance routes are depicted: enhanced glymphatic and meningeal-lymphatic drainage of soluble amyloid-β (Aβ) [44] and microglial recruitment with phagocytosis of Aβ deposits [45]. The evidence tier is marked on the panel itself: rodent models for both clearance mechanisms [44,45], and small open-label human series for BBB opening alone (five patients [46]; six patients receiving 17 treatments [47]). Reduction of amyloid signal in humans has not been shown to translate into clinical benefit. (**B**) Sonogenetics: a minimally invasive stimulation modality built upon an invasive genetic intervention. The paradigm is drawn as two separate steps with different symbols because their invasiveness differs. Step 1 (invasive and currently irreversible): delivery of the actuator by viral vector, through intracranial injection or high-dose systemic adeno-associated virus, with tropism, promoter-dependent specificity, immunogenicity, and long-term transgene expression as unresolved problems [140]. Step 2 (non-invasive): the ultrasound exposure itself. Three actuator classes are shown together with their distinct physics: the mutant bacterial channel MscL (for example, MscL-G22S and MscL-I92L), gated by membrane deformation [55,56,135]; exogenous hsTRPA1, gated mechanically in mammalian cells [139]; and TRPV1 in sonothermogenetics, which is gated by a focal temperature rise measured by magnetic resonance thermometry and is therefore a thermal rather than a mechanical mechanism [15]. TRP-4 in Caenorhabditis elegans is shown as the founding demonstration [54,134]. Evidence note: the descriptor “non-invasive” applies to the ultrasound exposure only and not to the paradigm as a whole. Figure concepts were derived from refs [15,44,45,46,47,54,55,56,134,135,139,140]. Created with BioRender.com. Created in Biorender. Giovanni Luca Cipriano. (2026) https://BioRender.com/jk5wjt9.

These transformative approaches, from the macro-scale mechanical clearance of amyloid aggregates to the nanoscale genetic engineering of acoustically sensitive circuits, illustrate the breadth of sonobiology as a preclinical research program (Figure 4). Their clinical standing differs markedly, however, and is set out in Section 8 and in Table 2.

## 8. Limitations and Barriers to Clinical Translation

The evidence summarized above is not of uniform weight, and the distinction is decisive for any claim of therapeutic potential. Four tiers should be kept separate. Cell-culture findings establish that a mechanical stimulus can alter a molecular readout, but they are obtained in media and under coupling conditions that do not reproduce the acoustic environment of tissue, and more than half of the in vitro work cited here was performed in non-neural lines (Table 1, Table 2 and Table 3). Animal in vivo outcomes add physiological context but, in this literature, the control that is applied least consistently is a manipulation or exclusion of the auditory pathway rather than a sham: sham or no-stimulation groups, and in several studies a control matched for the background acoustic noise of the device, are present more often than not [33,34,42,43]. Auditory controls are not confined to the studies designed to test the confound [48,49,50]: a sonothermogenetic study selected a pulse-repetition frequency outside the mouse hearing range and smoothed the onset and offset of every stimulus for that purpose [15], and a shockwave study excluded an auditory account of its motor responses by stimulating non-target body regions and by demonstrating energy dependence and an absence of movement in dead animals [53]. Where such controls are absent, the auditory route remains open (Section 3.6); and most in vivo studies are additionally reported for a single exposure setting, so neither the dose–response relationship nor the mechanism is constrained. Human feasibility studies demonstrate that a procedure can be performed safely in a small cohort; the Alzheimer’s disease work consists of an open-label phase I series of five and six patients [46,47]. At this point, ablative and non-ablative uses of acoustic energy must be separated because their clinical standing is not the same. Ablative magnetic resonance-guided high-intensity focused ultrasound has been evaluated in randomized, sham-controlled trials with prespecified clinical endpoints: unilateral thalamotomy reduced hand tremor relative to a sham procedure in 76 patients with essential tremor randomized 3:1, with a between-group difference of 8.3 points on a 32-point tremor scale at three months [141], and unilateral pallidotomy produced a response—improved motor function in the off-medication state or reduced dyskinesia—in 69% of patients with Parkinson’s disease against 32% after a sham procedure, in 94 patients randomized 3:1 [142]. That evidence establishes the efficacy of a thermal lesion placed under image guidance, and it does not transfer to the regimes that are the subject of this review. For low-intensity neuromodulation, microbubble-mediated blood–brain barrier opening, shockwaves, vibrotactile and audible stimulation, and acoustic neuroregeneration, no randomized, sham-controlled trial with a prespecified functional endpoint is present among the sources included here; the absence of such a trial from an included set is not in itself proof of its absence from the wider literature, and the statement is therefore made about this evidence base rather than about the field as a whole. It follows that reduced amyloid burden on positron emission tomography, increased GAP-43 immunoreactivity, and increased neurite length in culture are biomarkers and morphological surrogates, not interchangeable measures of durable functional recovery, and they are not treated as such in this review.

Several physical and safety barriers stand between preclinical demonstration and clinical use, and they differ by energy class. Transcranial delivery is limited by the skull, which attenuates, scatters, and aberrates the beam in a frequency-dependent and donor-specific manner; attenuation rises steeply with carrier frequency, which pushes human protocols toward 200–650 kHz and correspondingly larger focal volumes, and individual computed-tomography-based aberration correction is required for millimeter-scale targeting [85]. Thermal safety is not guaranteed by a low-intensity label: heating scales with ISPTA, duty cycle, and exposure duration. It is amplified at bone interfaces and under reduced perfusion and should be evaluated against the thresholds and reporting requirements set out in the ITRUSST biophysical safety consensus [16]. Cavitation introduces a separate, threshold-dependent risk: mechanical index, microbubble dose, and pulse length jointly determine whether oscillation remains stable or becomes inertial, and inertial cavitation during BBB opening can cause microhemorrhage and endothelial injury [13,87]. Vascular effects are an intended mechanism in ESWT and in FUS-mediated BBB opening and an unmonitored confound elsewhere, since changes in perfusion alter both drug delivery and the interpretation of hemodynamic imaging readouts [51]. Repeated-exposure safety is poorly characterized: the available human transcranial ultrasound safety data are dominated by data from a single or a few sessions and by retrospective symptom reporting [100,143], and no long-term repeated-exposure dataset exists for chronic neurological use.

Sonogenetic approaches carry an additional class of barriers that is not acoustic at all. Expression of the actuator must be achieved in the correct cell population at a level sufficient for ultrasound sensitivity and maintained for the duration of treatment, which raises questions of vector tropism, promoter specificity, immunogenicity, and reversibility that are independent of the stimulation itself [140]. Neither the long-term consequences of constitutive mechanosensitive or thermosensitive channel overexpression in neurons nor the behavior of these constructs under repeated sonication has been characterized.

Progress therefore depends less on the discovery of new mechanisms than on dosimetric and methodological discipline. Five requirements follow directly from the problems identified in this review: standardized reporting of exposure parameters in the ITRUSST format, including the intensity metric and whether it was measured in free water or estimated in situ [84]; in situ rather than free-water dose estimation for all transcranial work [85]; sham transducers matched for audible output together with deafened or masked control groups in every in vivo study claiming direct neuromodulation [48,49,50,92,93]; genetic or selective pharmacological identification of the transducing channel rather than calcium imaging alone, following the model of the astrocytic Trpa1 and Piezo1 studies [23,118]; and randomized, sham-controlled trials with prespecified functional endpoints before clinical efficacy is claimed for any non-ablative acoustic energy. Until these conditions are met, the non-ablative part of the field is best described as mechanistically promising and clinically unproven.

## 9. Conclusions

The integration of acoustics and neurobiology marks a profound shift from purely electrochemical models to a mechanobiological understanding of neural networks. Acoustic waves, ranging from low-frequency vibration to targeted focused ultrasound, deliver precise mechanical forces that are decoded by the cellular mechanome. By activating primary transducers such as Piezo1 and TRP channels, acoustic energy engages neurotrophic signaling (mTOR and p38 MAPK pathways) to support neurite regeneration and modulate glial phenotypes toward neuroprotective and reparative states; a continuous mechanistic axis extending through the cytoskeletal tensegrity network, the LINC complex, and chromatin remodeling remains, at this stage, an integrative hypothesis rather than a demonstrated pathway. The paradigm of “acoustic pharmacology” demonstrates that cellular responses are exquisitely tuned to specific frequencies and waveforms, offering a highly customizable therapeutic approach. As technologies like sonogenetics and targeted blood–brain barrier opening protocols advance to clinical frontiers, acoustic neuromodulation offers a tunable and largely non-invasive experimental strategy whose clinical value in neurodegenerative disease and traumatic nervous system injury remains to be established. The principal obstacles are evidentiary rather than conceptual: dosimetric reporting in metrics that cannot be converted into one another and whose free-water or in situ basis is frequently left implicit, an auditory confound that few in vivo studies control for, attribution of mechanism from calcium imaging alone, and the absence of randomized, sham-controlled human data for any non-ablative regime, in contrast with the ablative use of high-intensity focused ultrasound, which has been tested in that way (Section 8). Progress will be measured by the resolution of these issues rather than by the addition of further candidate mechanisms.

## 10. Methods

### 10.1. Search Strategy and Database Selection

This article is a narrative review, and it is deliberately not presented as a systematic one. No protocol was registered, no formal screening of records against prespecified eligibility criteria was carried out, and the synthesis is interpretive rather than protocol-driven; the breadth of the field, which spans acoustic physics, cell mechanobiology, neurobiology, and clinical neurology, makes a single prespecified question of the kind a systematic review requires impracticable. What is reported below is therefore the search procedure that was actually used, set out in sufficient detail to be reproduced and appraised. The literature search was primarily conducted using PubMed/MEDLINE to identify peer-reviewed studies concerning neurobiology, glial phenotypes, mechanosensitive ion channels (e.g., Piezo1, TRPV4), and translational neurology. To capture the multidisciplinary nature of acoustic mechanobiology and ensure exhaustive retrieval of high-impact emerging literature, the AI-powered academic search engine Consensus (https://consensus.app/) was integrated into the workflow. Consensus was specifically employed for advanced semantic querying to identify overlapping research areas—such as the intersection of acoustic physics, sonogenetics, and neurotrophic signaling—and to ensure that critical, newly published studies from 2024 and 2025 were not omitted. Database retrieval was completed by backward and forward citation chasing of every included primary study, so that the reference lists of the key sources and the literature citing them were examined in addition to the database output. The final search was run on 15 September 2026. The following concept blocks were combined with the Boolean operator AND, with terms within each block combined with OR. Block 1, exposure: “ultrasound”, “low-intensity pulsed ultrasound”, “LIPUS”, “focused ultrasound”, “LIFU”, “HIFU”, “transcranial ultrasound stimulation”, “shockwave”, “extracorporeal shockwave”, “audible sound”, “sound stimulation”, “vibration”, “vibrotactile”, “sonogenetics”. Block 2, mechanism: “mechanotransduction”, “mechanosensitive ion channel”, “Piezo1”, “Piezo2”, “TRPV4”, “TRPA1”, “TREK”, “LINC complex”, “YAP”, “chromatin remodeling”, “calcium signaling”. Block 3, target: “neuron”, “neurite”, “astrocyte”, “microglia”, “oligodendrocyte”, “Schwann cell”, “BDNF”, “GAP-43”, “neuroregeneration”, “blood–brain barrier”. Language and date filters were applied as described in Section 10.2.

### 10.2. Inclusion and Exclusion Criteria

The selection of articles was based on strict inclusion criteria: (1) peer-reviewed original research articles, comprehensive reviews, and clinical trials; (2) studies published in the English language; and (3) a primary publication timeframe spanning from 2010 to the date of the final search. Two sources published before 2010 were admitted as explicit exceptions to this window on the stated ground that each is the original and still-standard statement of a concept on which the argument depends and for which no later equivalent exists and that citing a secondary source in its place would have weakened the attribution. These are the canonical accounts of calcium-signaling dynamics, homeostasis, and remodeling, used in the Sources and Interpretation of Acoustically Evoked Ca^2+^ Signals section to separate a plasticity-range Ca^2+^ transient from a stress-range one [57], and the first demonstration of reversible, image-guided blood–brain barrier opening with focused ultrasound and microbubbles, which defines the regime discussed in Section 3.3 and Section 7 [89]. No other cited source predates 2010. The inclusion of pivotal studies from 2010 established the foundational molecular mechanisms of nucleosome dynamics and mechanical signaling, while the majority of the selected literature was published between 2019 and 2025 to reflect the latest advancements in focused ultrasound and sonogenetics. Articles lacking rigorous peer review, non-academic web sources, and studies primarily focused on purely auditory (cochlear) perception without non-auditory mechanotransductive pathways were excluded. Studies of auditory and vibrotactile stimulation were included specifically when they addressed molecular signaling, neuronal plasticity, or glial responses relevant to the mechanotransductive scope of this review. Because the review is narrative rather than systematic, records were appraised iteratively as the argument developed, and no screening log of records retrieved, de-duplicated, screened, and excluded was maintained; we therefore do not report study-selection counts and present no selection flow diagram, since either would imply a protocol-driven process that was not followed. The 144 sources cited in this review constitute the final included set; their composition by study design and by acoustic energy class is reported in Table 2 and Table 3, and the exposure characteristics of the representative primary studies are reconstructed in Table 1.

### 10.3. Data Extraction and Synthesis

Following the initial retrieval, full-text articles were evaluated to isolate specific wave parameters (frequency, waveform, intensity) and their corresponding molecular effects. The extracted data were synthesized and categorized into a hierarchical framework: (1) physical principles of acoustic energy; (2) molecular transducers and nuclear mechanotransduction; (3) neurotrophic and glial phenotypic shifts; and (4) translational clinical applications. This structured synthesis provided the basis for the narrative flow and the development of the mechanistic models presented in this review. Where sources disagreed, the discrepancy was resolved by weighting the evidence rather than by averaging it, using the following order of precedence: (1) studies in acoustically stimulated neural cells or tissue in which the transducing mechanism was identified genetically or with selective pharmacology; (2) studies in acoustically stimulated neural preparations with complete exposure reporting but attribution based on imaging or non-selective pharmacology; (3) studies in non-neural cells or using non-acoustic mechanical paradigms; and (4) narrative reviews and consensus statements. Where a conflict could not be resolved on these grounds—as for the auditory confound in in vivo ultrasound neuromodulation—both positions are reported, and the disagreement is stated explicitly in the text (Section 3.6). Exposure parameters were extracted verbatim from the primary source, retaining the metric as reported (ISPPA, ISPTA, ISATA, peak negative pressure, energy flux density, or sound pressure level) and recording free-water versus in situ measurement where stated; parameters absent from the source are marked “NR” in Table 1 and were not imputed. For Table 1, exposure parameters and control conditions were subsequently re-extracted for every included primary study from the full text and, where available, the supplementary material rather than from the abstract; where these differ, the full text and supplementary material take precedence. Published corrections and errata to the primary sources were checked and are reflected in the table. Parameters inapplicable to the exposure—such as a pulse-repetition frequency for continuous vibration, audible sound, or a shockwave—are marked “N/A”. Reporting of this narrative review was guided by the SANRA scale for the quality assessment of narrative review articles [144].

## Figures and Tables

**Table 1:** Reconstructed exposure and evidence table for representative studies cited in this review. Parameters were re-extracted from the primary sources and are reported with the metric used by the original authors. Three cases are distinguished rather than merged. “NR” denotes a parameter absent from the full text and supplementary material of the source and is not used for a parameter the source reports outside its abstract. “N/A” denotes a parameter that does not apply to the exposure, such as a pulse-repetition frequency for continuous vibration, for audible sound, or for a shockwave. Where a source enumerates its exposures as a parameter set in supplementary tables rather than as a single protocol, the entry cites those tables; where a published value is internally inconsistent with another parameter in the same source, it is reproduced as printed, and the inconsistency is noted rather than silently amended. As before, a numerical value that the source reports without restating the averaging convention beside it is preserved, with the convention taken from the Methods section of the same source and annotated as such. In neither case is an absent parameter imputed from the device or from a companion publication. Intensity metrics are given with the descriptor used by the authors (ISPPA, spatial-peak pulse-average; ISPTA, spatial-peak temporal-average; ISATA, spatial-average temporal-average; ISAI, spatial-average intensity; EFD, energy flux density; SPL, sound pressure level referenced to 20 µPa) and are annotated as free-water where the source states that the measurement was made in water. Focusing and intensity are reported in separate columns because they are orthogonal parameters (Section 3.1). The control column reports four categories separately because each licenses a different inference: a sham or no-stimulation control, which establishes that an effect exists; a control matched for background acoustic noise, which constrains a purely auditory account of an effect obtained with a mechanically noisy device; a manipulation of auditory function (cochlear ablation, chemical or genetic deafening, acoustic masking, or smoothing of the stimulus envelope), which is what separates direct neuromodulation from auditory-pathway activation (Section 3.6); and a comparison across frequencies, waveforms, or exposure levels, which is what licenses a claim that a parameter is optimal. No category is inferred from the presence of another. PRF, pulse-repetition frequency; DC, duty cycle; CW, continuous wave; DRG, dorsal root ganglion; BBB, blood–brain barrier; MRgFUS, magnetic resonance-guided focused ultrasound.

Study [Ref.]	Energy Class	Model System	Carrier Frequency	PRF/Pulse Duration/Duty Cycle	Exposure Schedule	Intensity or Pressure (Metric as Reported)	Focusing	Microbubbles	Control Conditions Reported (Sham or No-Stimulation/Background Sound/Auditory-Pathway Manipulation/Frequency, Waveform, or Level Comparison)	Principal Outcome	Level of Evidence and Principal Limitation
Kumeta 2018 [31]	Audible sound	ST2 murine stromal; C2C12; NIH3T3 (non-neural)	440 Hz (55, 110 Hz, 4 kHz also tested)	N/A—continuous audible sound, not a pulsed waveform	1–4 h continuous; some 1 h + silent chase	94.0 dB SPL ≈ 1.00 Pa re 20 µPa (76–88 dB also tested)	Unfocused (airborne)	No	Sham yes (silent control); background sound N/A (in vitro); auditory manipulation N/A; frequency and waveform comparison yes (55, 110, 440 Hz, and 4 kHz; sine, triangle, square)	Suppression of Ptgs2, Ctgf, Tnc mRNA; cell-type-dependent	In vitro, non-neural; not replicated in neurons or glia
Kumeta 2025 [32]	Audible sound	C2C12 murine myoblasts (non-neural)	440 Hz sine; 14 kHz sine; white noise	N/A—continuous acoustic emission, not a pulsed waveform	2 h and 24 h continuous emission; RNA-seq at both time points	100 Pa (10–250 Pa tested); measured in water with a calibrated hydrophone	Unfocused; vibrational transducer coupled directly to the medium	No	Sham yes (silent control); background sound N/A; auditory manipulation N/A; frequency and waveform comparison yes (440 Hz, 14 kHz, white noise; sine, triangle, square)	42 early (2 h) and 145 late (24 h) differentially expressed genes; FAK-dependent Ptgs2 induction and prostaglandin E2 release; adhesion area +15–20% in 1 h	In vitro, non-neural; single laboratory; fluid motion not fully separable from the compressional wave
Kumeta 2025 [32]	Audible sound	3T3-L1 murine preadipocytes (non-neural)	440 Hz sine	N/A—continuous acoustic emission, not a pulsed waveform	During adipogenic induction only (methylisobutylxanthine, dexamethasone, and insulin): continuous days 0–3 (72 h), or days 0–1, 1–2, or 2–3 (24 h each), or 2 h/day × 3; readout at day 3 and day 7	100 Pa	Unfocused; transducer coupled directly to the medium	No	Sham yes (silent control); background sound N/A; auditory manipulation N/A; frequency comparison no (440 Hz sine only); schedule comparison yes	Cebpa and Pparg suppressed at day 3 (to 0.18 and 0.26 of control with continuous days 0–3); undifferentiated fraction 0.23 → 0.39 and 0.43; lipid accumulation −13–15%	In vitro, non-neural; stimulation not applicable after day 4 because differentiating cells detach; single laboratory
Suk 2023 [33]	Vibrotactile	Nine-month-old male Tau P301S mice and six-month-old female CK-p25 mice; four-month-old C57BL/6J mice for the c-Fos experiment. Continuous 40 Hz sinusoid, not a pulsed waveform	40 Hz	N/A—continuous 40 Hz sinusoid, not a pulsed waveform	1 h/day; 21 d (P301S) or 42 d (CK-p25)	≈16 µm peak displacement (accelerometer)	Whole-body contact	No	Sham yes (no-stimulation group in identical cages on speakers not connected to the amplifier); background sound yes (powered and unpowered speakers alternated in the room so that both groups received comparable humming noise); auditory manipulation no; frequency comparison no (40 Hz only)	↓ pTau, ↓ DNA damage, ↓ neuronal loss; ↑ motor function	Animal in vivo; the vibration-free control is matched for background acoustic noise, so a purely auditory account is constrained, but the mechanical and somatosensory routes are not dissociated; 40 Hz was not titrated against neighboring frequencies, which the authors state explicitly
Liu 2025 [34]	Whole-body vibration	Aged male Sprague–Dawley rats	40 Hz (20 and 80 Hz also tested during electrocorticogram recording; 80 Hz comparator for the two-month protocol)	N/A—continuous sinusoidal vibration at a fixed frequency, not a pulsed waveform	15 min twice daily, 5 d/week, 2 months	0.8 mm vertical amplitude	Whole-body	No	Sham yes (sham-WBV group on a replaced vibration plate, without actual vibration); background sound yes (movement sounds simulated for the sham group); auditory manipulation no; frequency comparison yes (20, 40, and 80 Hz for electrocorticography; 40 vs. 80 Hz for the two-month protocol; young-rat age control)	40 Hz gamma oscillations; ↑ spatial memory; ↓ CA1 apoptosis	Animal in vivo; both 40 and 80 Hz reduced hippocampal inflammatory markers, so that effect is attributed by the authors to vibration rather than to gamma entrainment; the platform was electromagnetically isolated during electrocorticography but not during the two-month training
Grosman-Dziewiszek 2022 [35]	Nanovibration	SH-SY5Y human neuroblastoma line	40 Hz vs. 100 Hz	N/A—continuous sinusoidal drive, not a pulsed waveform	5 days	Displacement amplitude 14–44 nm across the culture plate, corresponding to accelerations of 0.6–1.7 mm/s^2^, measured by scanning laser vibrometry; 1 V RMS continuous sinusoidal drive. The figure of <1.0 g quoted previously is the literature definition of low-magnitude, low-frequency vibration given in that paper’s introduction, not a measured value	Plate-coupled	No	Sham yes (unstimulated control); background sound N/A; auditory manipulation N/A; frequency comparison yes (40 vs. 100 Hz)	40 Hz > 100 Hz for neurite length and proliferation	In vitro immortalized line; not primary neurons
Iaccarino 2016 [36]; Martorell 2019 [37]	Sensory gamma. [36] 2016: visual 40 Hz light flicker only, no auditory stimulation. [37]: auditory 40 Hz tone trains and combined auditory plus visual	[36] 5XFAD and APP/PS1 mice, with a separate optogenetic FS-PV arm. [37]: 5XFAD and APP/PS1 mice and the Tau P301S tauopathy model	40 Hz envelope	40 Hz	1 h/day for 7 days in both studies	N/A (no mechanical dose delivered to the brain)	N/A	No	Sham yes (no-stimulation control); background sound not separable from the stimulus, which is itself sensory; auditory manipulation no; frequency comparison yes (non-40 Hz and random-stimulation controls)	↓ Aβ; microglial morphology change; ↑ cognition	Afferent-pathway mechanism, not direct mechanotransduction. The outcomes differ by study, modality, and region and are not interchangeable: Iaccarino 2016 reports reduced Abeta40 and Abeta42 in the visual cortex and reduced plaque load in aged mice, with microglial morphological transformation, and reports no cognitive outcome; Martorell 2019 reports improved spatial and recognition memory, reduced amyloid in the auditory cortex and hippocampus, reduced phosphorylated tau in the P301S model, and a reduction in the medial prefrontal cortex that required combined auditory and visual stimulation and was obtained with neither modality alone
Han 2020 [38]	LIPUS (unfocused)	Rat DRG explants, axotomy	1.5 MHz	PRF 10 or 100 Hz; 200 ms sonication; DC 20%	10 min once, immediately after axotomy	ISPTA 25 mW/cm^2^ (free water, calibrated needle hydrophone); the source gives the value as acoustic intensity in the Figure 1 legend and defines acoustic intensity as ISPTA in its Methods section	Unfocused	No	Sham yes (non-stimulated axotomized control); background sound N/A; auditory manipulation N/A; pulse-repetition-frequency comparison yes (10 vs. 100 Hz)	↑ neurite number and length; ↑ mTOR; ↑ GAP-43 (10 Hz > 100 Hz)	In vitro explant; single intensity level; no functional-recovery endpoint
Iacoponi 2023 [39]	LIPUS	Human U937-derived M1-like macrophages	38 kHz, 1 MHz, and 5 MHz compared	Duty cycles of 10%, 20%, 30%, and 40% compared	30, 60, 90, and 120 min compared (full factorial with the above)	25, 100, 250, and 450 mW/cm^2^; transducers previously characterized by pressure field maps and intensity calibration	Unfocused	No	Sham yes (unstimulated control); background sound N/A; auditory manipulation N/A; frequency and level comparison yes (38 kHz, 1 MHz, 5 MHz; 25–450 mW/cm^2^)	↓ IL-1β, IL-8, TNF-α; p38 MAPK activation	In vitro, non-neural immune line
Hsu 2023 [40]	LIPUS	BV-2 murine microglia; LPS (M1)/IL-4 (M2)	1 MHz	PRF 100 Hz; 2 ms burst; DC 20%	3 × 5 min with 5 min intervals (15 min total)	ISAI 30 mW/cm^2^ (free water, degassed)	Unfocused	No	Sham yes (unstimulated LPS- and IL-4-treated controls); background sound N/A; auditory manipulation N/A; frequency comparison no (1 MHz only)	↓ M1 (iNOS, TNF-α, STAT1); ↑ M2 (PPARγ)	In vitro cell line; no in vivo confirmation
Li 2024 [41]	LIPUS	CX3CR1-GFP mice (two-photon imaging) and Sprague–Dawley rats (electrophysiology and histology; n = 4 LIPUS, n = 4 control), intracortical microelectrode implantation	1.13 MHz	PRF 2 Hz; 22 ms burst duration; DC 4.4%	3 × 5 min with 5 min gaps per session; daily for one week after implantation, then twice weekly for five weeks; imaging and recording to day 28	≈300 mW/cm^2^ ISPTA (hydrophone in degassed water, measured through the imaging coverslip)	Unfocused	No	Sham yes (sham treatment of matched duration with the transducer mounted but not driven; separate untreated control cohort); background sound NR (all animals lightly anesthetized during treatment); auditory manipulation NR; frequency comparison no (single parameter set, which the authors identify as a limitation)	↓ microglial activation and probe encapsulation; improved recordings	Single exposure setting; no parameter dissection
Li 2025 [42]	LIPUS	C57BL/6J mice; STZ + high-fat-diet diabetic neuropathy	1 MHz	DC 20%; PRF NR	20 min/day, 5 d/week, 56 days	ISATA 200 mW/cm^2^	Unfocused; eight flat circular probes of 25 mm diameter, the treated region fully covered by one probe	No	Sham no sonication sham; randomized allocation to an untreated diabetic control group, with a non-diabetic sham-model group as reference; background sound NR; auditory manipulation N/A; frequency comparison no (1 MHz, single dose level)	Improved nerve fiber morphology; ↓ MDA; Keap1/Nrf2/HO-1 change	Single dose level; no dose–response
Qiu 2019 [20]	LIFU	HEK293T (Piezo1-transfected); mouse primary cortical neurons	500 kHz	PRF 1 kHz; DC 40%; 200 tone bursts per stimulus	Per-stimulus trains; 20 min incubator exposures	Peak acoustic pressure 0.1–0.5 MPa as a dose–response series; 0.3 MPa threshold for significant Ca^2+^ influx in neurons	Unfocused—triangular waveguide coupled at 45 degrees beneath the dish with an acoustic absorber; field mapped by a needle hydrophone on a 3D stage	No	Sham yes (unstimulated control); background sound N/A; auditory manipulation N/A; frequency comparison no (500 kHz only); pressure comparison yes; genetic and pharmacological controls yes (Piezo1 knockdown, GsMTx-4)	Piezo1-dependent Ca^2+^, c-Fos, p-CaMKII, p-CREB	Knockdown and GsMTx-4 only partial; no patch-clamp
Yoo 2022 [30]	FUS (low intensity)	Cultured rat/mouse cortical neurons (GCaMP6f/Ace2N)	300 kHz (670 kHz tested)	CW; 1 and 1.5 kHz PRF tested; 0–500 ms pulses; DC 100%	Single pulses, 20 s inter-pulse interval	0–15 W/cm^2^ ISPPA (free water)	Focused	No	Sham yes (unstimulated control); background sound N/A; auditory manipulation N/A; frequency comparison yes (300 and 670 kHz; 1 and 1.5 kHz PRF)	Mechanosensitive Ca^2+^ entry upstream of voltage-gated amplification	In vitro; no in vivo circuit validation
Oh 2020 [23]	LIFU	Mouse cortex in vivo; acute slices and cultured astrocytes; astrocyte-specific and global Trpa1 knockout. Three separate exposure configurations are reported in the STAR Methods section and are not interchangeable	350 kHz (transcranial tail-movement test); 500 kHz (multielectrode-array recording); 430 kHz (in vitro Ca^2+^ imaging; 500 um transducer, measured resonant frequency 430 kHz)	350 kHz config: PRF 1.5 kHz (inter-pulse interval 0.67 ms), pulse duration 0.23 ms. 500 kHz config: PRF 1.16 kHz, pulse duration 20.97 ms as printed (internally inconsistent with the stated PRF, which implies a 0.86 ms interval; reproduced as published, not amended). 430 kHz config: PRF 2 kHz, pulse duration 0.25 ms. Duty cycles not stated by the source	Sonication duration 66.67 ms (350 kHz, transcranial); 100 ms (430 kHz, in vitro)	0.7020 W/cm^2^ (350 kHz, transcranial), one point of a six-point series of 0.2570, 0.4410, 0.7020, 1.1530, 2.0500, and 4.000 W/cm^2^ (Figure S1C); spatial-peak pressure 11.52 kPa (500 kHz, MEA); 306.1 mW/cm^2^ at 66 V (430 kHz, in vitro), transducer controllable 10.2–67.3 kPa over 11–66 V	Focused	No	Sham yes (unstimulated and non-transgenic controls); background sound NR; auditory manipulation no; genetic control yes (astrocyte-specific and global Trpa1 deletion with TRPA1 re-expression); pharmacological control yes; intensity comparison yes (six-point series); skull attenuation measured ex vivo by a calibrated needle hydrophone in a degassed water tank and not significantly different across the 30–500 mW/cm^2^ range (Figure S1B,C)	LILFU activates astrocytes via TRPA1, with Best1-mediated glutamate release acting on neuronal NMDA receptors; a published Correction (Curr Biol 2020;30:948) withdraws the stronger claim that TRPA1 is itself directly gated by ultrasound [23]. Ultrasound-evoked Ca^2+^ and neuronal activation	Strong genetic attribution. Exposure is fully reported but differs in carrier frequency, pulse structure, and intensity between the in vivo, multielectrode-array, and in vitro experiments, so the three results rest on three different exposures; the auditory confound is not addressed; the direct-gating claim is withdrawn by the authors’ own Correction
Bobola 2020 [43]	Transcranial FUS	5XFAD mice	2.0 MHz	PRF 40 Hz; 400 µs pulses	5 s on/5 s off, 1 h/day, 5 days	ISPPA 190 W/cm^2^; ISPTA 3.0 W/cm^2^	Focused	No	Sham NR; background sound NR; auditory manipulation no; frequency comparison no (single protocol)	≈47% reduction in Aβ plaque load; acute microglial activation	Single exposure setting with no parameter dissection and no manipulation of the auditory pathway, although age-matched sham-exposed animals were used in both arms and the ambient 40 Hz field was measured directly at both the treatment and the sham position with the transducer on and off and was in fact louder at the sham position. The sustained plaque reduction and the acute microglial response were not concurrent
Lee 2020 [44]	FUS + microbubbles (BBB opening)	5XFAD mice ± deep cervical lymph node ligation	715 kHz	PRF 1 Hz; 20 ms bursts; DC 2%	60 s sonication per session; once weekly for six weeks (non-ligated) or for four weeks after deep cervical lymph node ligation	0.15 W acoustic power; peak negative pressure 0.42 MPa in water	Focused	Yes	Sham no (untreated naïve 5XFAD mice and wild-type littermates as controls; no transducer-off sham sonication described); background sound NR; auditory manipulation NR; frequency comparison no (715 kHz only)	↑ CSF Aβ drainage via meningeal lymphatics; improved memory	Single transgenic model; no human translation
Karakatsani 2023 [45]	FUS + microbubbles	3xTg-AD mice (14–16 mo) and AD/MCI patients	1.5 MHz (mice); 0.25 MHz (patients)	10 ms pulse length; PRF 5 Hz (mice), 2 Hz (patients)	Mice: once weekly for four consecutive weeks (first cohort) or a single sonication (second cohort); patients: 2 min treatment per session	Derated peak negative pressure 0.40 MPa (mice, after correction for 18% murine skull attenuation); 200 kPa derated, mechanical index 0.4 (patients)	Focused	Yes	Sham yes (identical procedure with the transducer not triggered, matched microbubble injection, and matched anesthesia duration; random assignment, 11 mice per group), plus age- and sex-matched non-transgenic controls; background sound N/A; auditory manipulation N/A; frequency comparison no	↓ amyloid and tau; ↑ spatial memory in mice; safe in patients	Mixed preclinical/early clinical; no efficacy endpoint
Lipsman 2018 [46]	MRgFUS + microbubbles	Carrier 220 kHz (ExAblate Neuro, 1024-element array); bursts of 2 ms on and 28 ms off over 300 ms at a 2.7 s repetition interval, duty cycle 0.74%; mean maximum acoustic power 4.6 W (stage 1) and 4.5 W (stage 2), delivered at 50% of the per-target cavitation threshold established by a ramp test; intensity in W/cm^2^ not reported. 5 patients, early to moderate Alzheimer’s disease	NR	NR	Repeated sonications, two stages	NR	Focused	Yes	Sham no (open-label, uncontrolled); background sound N/A; auditory manipulation N/A; frequency comparison no	Safe and reversible BBB opening in all patients with closure by 24 h; no serious adverse events; no clinically significant cognitive change at 3 months; florbetaben SUVr change −0.14 +/− 0.22 after stage 1 and −0.08 +/− 0.21 after stage 2, neither significant	Open-label phase I, n = 5; not designed to show efficacy
Rezai 2020 [47]	MRgFUS + microbubbles	6 patients with Alzheimer’s disease (17 treatments)	220 kHz	NR	3 sessions, 2 weeks apart	NR	Focused	Yes	Sham no (open-label case series); background sound N/A; auditory manipulation N/A; frequency comparison no (220 kHz only)	Reversible hippocampal BBB opening, 14–71% of hippocampal volume	Open-label, prospective phase II trial without a control arm; not designed to demonstrate efficacy and no clinical endpoint
Sato 2018 [48]; Guo 2018 [49]	Transcranial FUS	Mice. Thy1-GCaMP6s transgenic males >20 weeks with thinned skull for wide-field calcium imaging; C57BL/6J males 8–12 weeks for electromyography. Carrier 500 kHz; PRF 1.5 kHz; pulse duration 200 us (100 cycles); 120 pulses; duty cycle 30%; stimulus approximately 80 ms; ISPTA 0.034–4.2 W/cm^2^ in five steps, calibrated by a fiber-optic hydrophone in free water	NR	NR	NR	NR	Focused	No	Sham NR; background sound NR; auditory manipulation yes (cochlear ablation; chemical deafening); frequency comparison NR	The auditory cortex activated earliest and at the lowest intensities, with a contralateral bias consistent with an inner-ear route; the cortical pattern closely resembled that evoked by audible sound and was unlike that evoked by a light flash. Chemical deafening (kanamycin plus furosemide) reduced electromyographic responses to both ultrasound and audible sound while leaving air-puff responses intact, and uncoupling the transducer abolished the motor response. In guinea pigs, bilateral transection of the auditory nerves or bilateral removal of cochlear fluids eliminated ultrasound-evoked activation of the auditory and somatosensory cortex across the full parameter range (220 kHz carrier; 25–2000 kPa; pulse-repetition frequencies from single-pulse to 16 kHz); residual activation after transection was 0% of sites in most paradigms and at most 6.25%, in only one animal of three	Control studies; define the confound rather than the mechanism. No cochlear ablation was performed in either study. In the mouse study, chemical deafening could not be carried out in the older transgenic animals used for imaging because of high mortality, so no cortical data from deafened animals exist and the deafening result there is a motor result only. An erratum to the guinea-pig study corrects the proportion of the somatosensory cortex activated before removal of cochlear fluids to 61.5 +/− 7.2% (Neuron [49]
Mohammadjavadi 2019 [50]	Transcranial FUS	32 female mice aged 8–12 weeks (21 wild-type normal-hearing C57BL/6; 7 TRIOBP and 4 Samba LOXHD1 genetically deaf). Carrier 500 kHz, delivered from an unfocused planar transducer (Olympus V301) with waveguides, chosen by the authors in explicit contrast to focused transducers; four 80 ms envelopes at matched intensity (CW rectangular; pulsed 1.5 kHz PRF at 80% duty cycle; pulsed 8.0 kHz PRF; CW smoothed over 12 ms); ISPPA 1, 2.79, and 3.78 W/cm^2^, then a constant 2.9 W/cm^2^, from optical-hydrophone pressures in free water. Wild-type normal-hearing mice and two strains of genetically deaf mice; electromyography and auditory brainstem responses	NR	NR	NR	NR	Focused	No	Sham NR; background sound NR; auditory manipulation yes (two strains of genetically deaf mice; auditory brainstem responses recorded; smoothing of the stimulus envelope); frequency comparison no, envelope shape varied instead	Motor responses persist in the absence of auditory activation; smoothing the stimulus envelope removes the auditory brainstem response without altering the motor response	Counter-evidence to the auditory-confound interpretation
Sung 2025 [51]	ESWT	C57BL/6 mouse critical limb ischemia; HUVEC and C2C12	N/A (shockwave)	180 impulses per session	Days 1, 3, 5, and 7 after induction	EFD 0.15 mJ/mm^2^ (0.10–0.25 mJ/mm^2^ tested)	Focused extracorporeal shockwave probe (Duolith SD1, STORZ MEDICAL)	No	Sham yes (untreated ischemic control); background sound N/A; auditory manipulation N/A; energy comparison yes (0.10–0.25 mJ/mm^2^)	↑ perfusion and angiogenesis; ↓ inflammation via GPR120	Non-neural outcome; mechanism extrapolated to nerves
Park 2020 [52]	ESWT	Rat sciatic nerve crush; 97 rats in five groups (healthy non-injured control; crush with three ESWT sessions assessed at 3 and at 5 weeks; crush without ESWT assessed at 3 and at 5 weeks)	N/A	1500 impulses per session	3 sessions at weekly intervals	EFD NR	Focused	No	Sham no (no-ESWT crush groups at 3 and 5 weeks and a healthy non-injured group; no sham-probe application described); background sound N/A; auditory manipulation N/A; dose comparison no (single protocol; EFD not reported)	Myelination and myelin basic protein showed a non-significant trend toward an increase; print width and print area of the injured limb were significantly larger than in the no-ESWT groups	EFD not reported; the remyelination outcome is a trend rather than a significant effect; no molecular mechanism; no long-term follow-up
Ham 2024 [53]	Shockwave (deep brain modulation); dielectric-breakdown (DB) and electromagnetic (EM) sources compared	Mouse, healthy deep-brain target	N/A	N/A for pulse-repetition frequency in the ultrasound sense—this is a shockwave exposure. Pulse duration approximately 2000 ns; shocks delivered at 3 per second	15 stimulations per session at 3 per second	Peak pressure 5–60 MPa at the focal point; energy density 0.03–0.76 mJ/mm^2^; input energy 0.12–0.35 J. Motor-threshold series at 0.38, 0.09, and 0.03 mJ/mm^2^ gave 94%, 18%, and 0% response rates	Focused; focal zone approximately 3 × 6 mm (DB source) and 4 × 45 mm (EM source)	No	Sham yes (sham stimulation of non-target body regions, evoking no significant motor response); auditory manipulation yes—the authors verified that motor responses were not auditory artifacts of the shockwave noise by stimulating the front leg and thigh and by demonstrating energy dependence; an additional control in dead animals showed no movement at the highest energy density; energy comparison yes; source comparison yes (DB versus EM)	↑ hypothalamic activity and hippocampal neurogenesis; no spatial-memory gain; reduced locomotion	Mechanism inferred from numerical modeling, not measured
Ibsen 2015 [54]	Sonogenetics (TRP-4)	C. elegans; trp-4 mutants and misexpression transgenics	2.25 MHz	Single 10 ms pulse	One pulse per trial	Peak negative pressure 0–0.9 MPa (0.41–0.6 MPa used)	Focused	Yes	Sham yes (trp-4 mutants and non-transgenic controls); background sound N/A; auditory manipulation N/A; pressure comparison yes (0–0.9 MPa)	TRP-4-dependent reversal behavior and neuronal Ca^2+^ activation	Invertebrate; requires microbubbles; behavioral correlation only
Ye 2018 [55]	Sonogenetics (MscL-I92L)	Rat hippocampal neurons, primary culture	Approximately 29.92 MHz, measured by a network analyzer; generated by a pair of interdigital transducers on a surface-acoustic-wave chip	Stimulus durations of 25 ms, 50 ms, and 1 s; pulse trains at 1, 5, and 10 Hz; repeated activation at 100 ms intervals	NR	Peak negative pressure adjustable from 0.12 to 0.45 MPa as a dose–response series; activation threshold 0.25 MPa for I92L MscL; temperature rise at 0.45 MPa measured at <0.1 C	Unfocused—surface-acoustic-wave chip with interdigital transducers, not a focused transducer	No	Sham yes (neurons not expressing MscL-I92L); background sound N/A; auditory manipulation N/A; frequency comparison NR	Ultrasound-evoked action potentials in MscL-I92L neurons	In vitro; no circuit-level validation
Yang 2021 [15]	Sonothermogenetics (TRPV1)	Mouse striatal CaMKII neurons; HEK293T	1.7 MHz (in vitro); 1.5 MHz (behavior)	PRF 10 Hz; DC 40% (PW) or 100% (CW)	2 sonications per neuron, 80 s apart; 3–5 per mouse	Peak negative pressure 1.0–1.3 MPa (in vitro); 0, 0.9, 1.3 MPa (behavior)	Focused	No	Sham yes (0 MPa group and TRPV1-negative littermates); background sound NR; auditory manipulation yes—a 10 Hz PRF was chosen deliberately to lie outside the mouse hearing range and the onset and offset of every stimulus were smoothed in all groups, both explicitly to minimize auditory-pathway activation; pressure and duty-cycle comparisons yes; pharmacological and genetic controls yes (capsaicin, capsazepine, TRPV1-transduced versus non-transduced neurons)	TRPV1-dependent Ca^2+^ influx and contralateral rotation	Thermal, not mechanical, mechanism; single-session safety only
Xian 2023 [56]	Sonogenetics (MscL-G22S)	Mouse dorsal striatum, VTA, STN; Parkinson’s disease model	0.5 MHz (0.9 MHz also used)	PRF 1 kHz; 400–500 µs pulse width; DC 40–50%; 300 ms burst; a smoothed (Hann-windowed) 300 ms waveform was also used	Repeatable pulse trains	Peak pressure 0.05–0.2 MPa; modeled focal temperature rise <0.2 °C	Focused	No	Sham yes (EYFP-only and MscL-WT controls); background sound NR; auditory manipulation yes (c-Fos in the auditory cortex comparable between groups, and a smoothed Hann-windowed envelope was adopted after a place-preference assay indicated an auditory confound with rectangular waveforms); frequency and pressure comparison yes (0.5 and 0.9 MHz; 0.05–0.2 MPa)	↑ locomotion and dopamine release; improved motor coordination	Requires AAV delivery; the thermal contribution is excluded by numerical modeling rather than by in situ measurement

**Table 2:** Distribution of the 144 sources cited in this review by study design. Classification was taken from the PubMed publication type where available and otherwise from the study design described in the abstract; percentages are of the total reference list and are rounded to one decimal place, so they need not sum to exactly 100.

Study Design	n	% of 144
Review, consensus statement, or guideline	59	41.0
Animal in vivo study	37	25.7
In vitro study (cell line, primary culture, or explant)	27	18.8
Biophysical, computational, or in silico analysis	14	9.7
Human study (feasibility, safety, or observational)	5	3.5
Randomized controlled trial with a clinical endpoint	2	1.4
Total	144	100.0

**Table 3:** Distribution of the 144 sources cited in this review by acoustic energy class. Sources that supply molecular, mechanobiological, or methodological background without reporting an acoustic exposure are grouped separately because they constrain how far the mechanistic synthesis in Section 2 can be taken.

Acoustic Energy Class	n	% of 144
Focused ultrasound (LIFU, FUS, HIFU, microbubble-mediated BBB opening)	36	25.0
Audible sound and vibration (including vibrotactile and whole-body)	21	14.6
LIPUS and low-intensity unfocused ultrasound	19	13.2
Extracorporeal shockwave therapy	5	3.5
Sonogenetics and sonothermogenetics	6	4.2
Non-acoustic mechanobiological, molecular, or methodological background	57	39.6
Total	144	100.0

## Data Availability

No new data were created or analyzed in this study.

## Abbreviations

The following abbreviations are used in this manuscript:

AAV	Adeno-associated virus
AD	Alzheimer’s disease
ARF	Acoustic radiation force
Aβ	Amyloid-beta
BBB	Blood–brain barrier
BDNF	Brain-derived neurotrophic factor
CaMKII	Ca^2+^/calmodulin-dependent protein kinase II
CREB	cAMP response element-binding protein
CSF	Cerebrospinal fluid
CW	Continuous wave
DC	Duty cycle
DRG	Dorsal root ganglion
ECM	Extracellular matrix
EFD	Energy flux density
EMT	Epithelial-to-mesenchymal transition
ERK	Extracellular signal-regulated kinase
ESWT	Extracorporeal shockwave therapy
FAK	Focal adhesion kinase
FLIPUS	Focused low-intensity pulsed ultrasound
FUS	Focused ultrasound
FUS-MB	Focused ultrasound with microbubbles
GAP-43	Growth-associated protein 43
HIFU	High-intensity focused ultrasound
IDP	Intrinsically disordered protein
IL	Interleukin
iNOS	Inducible nitric oxide synthase
ISAI	Spatial-average intensity
ISATA	Spatial-average temporal-average intensity
ISPPA	Spatial-peak pulse-average intensity
ISPTA	Spatial-peak temporal-average intensity
ISWI	Imitation switch
ITRUSST	International Transcranial Ultrasonic Stimulation Safety and Standards
K2P	Two-pore-domain potassium
KASH	Klarsicht/ANC-1/Syne homology
LIFU	Low-intensity focused ultrasound
LINC	Linker of nucleoskeleton and cytoskeleton
LIPUS	Low-intensity pulsed ultrasound
LITUS	Low-intensity transcranial ultrasound stimulation
LTP	Long-term potentiation
MAPK	Mitogen-activated protein kinase
MBP	Myelin basic protein
MCI	Mild cognitive impairment
MDA	Malondialdehyde
MRgFUS	Magnetic resonance-guided focused ultrasound
MscL	Mechanosensitive channel of large conductance
mTOR	Mechanistic target of rapamycin
NF-κB	Nuclear factor kappa B
NGF	Nerve growth factor
NR	Not reported
OPC	Oligodendrocyte precursor cell
PET	Positron emission tomography
pFUS	Pulsed focused ultrasound
PI3K	Phosphoinositide 3-kinase
PLCγ	Phospholipase C gamma
PNP	Peak negative pressure
PPARγ	Peroxisome proliferator-activated receptor gamma
PRF	Pulse-repetition frequency
PSD95	Postsynaptic density protein 95
SANRA	Scale for the Assessment of Narrative Review Articles
SPL	Sound pressure level (referenced to 20 µPa)
STAT	Signal transducer and activator of transcription
SUN	Sad1/UNC-84
SYP	Synaptophysin
TEAD	TEA domain transcription factor
tFUS	Transcranial focused ultrasound
TLR4	Toll-like receptor 4
TNF-α	Tumor necrosis factor alpha
TrkB	Tropomyosin receptor kinase B
TRP	Transient receptor potential
TRPA1	Transient receptor potential ankyrin 1
TRPC1	Transient receptor potential canonical 1
TRPV1	Transient receptor potential vanilloid 1
TRPV4	Transient receptor potential vanilloid 4
VTA	Ventral tegmental area
YAP/TAZ	Yes-associated protein/transcriptional co-activator with PDZ-binding motif
